# Expression of receptor-type protein tyrosine phosphatase in developing and adult renal vasculature

**DOI:** 10.1371/journal.pone.0177192

**Published:** 2017-05-25

**Authors:** Keiko Takahashi, Rachel Kim, Colette Lauhan, Yuna Park, Nghiep G. Nguyen, Dietmar Vestweber, Melissa G. Dominguez, David M. Valenzuela, Andrew J. Murphy, George D. Yancopoulos, Nicholas W. Gale, Takamune Takahashi

**Affiliations:** 1 Division of Nephrology and Hypertension, Vanderbilt University School of Medicine, Nashville, Tennessee, United States of America; 2 University of Oklahoma College of Medicine, Oklahoma City, Oklahoma, United States of America; 3 Max-Planck-Institute of Molecular Biomedicine, Münster, Germany; 4 Regeneron Pharmaceuticals, Inc., Tarrytown, New York, United States of America; 5 Department of Cancer Biology, Vanderbilt University School of Medicine, Nashville, Tennessee, United States of America; National Cancer Institute, UNITED STATES

## Abstract

Renal vascular development is a coordinated process that requires ordered endothelial cell proliferation, migration, intercellular adhesion, and morphogenesis. In recent decades, studies have defined the pivotal role of endothelial receptor tyrosine kinases (RPTKs) in the development and maintenance of renal vasculature. However, the expression and the role of receptor tyrosine phosphatases (RPTPs) in renal endothelium are poorly understood, though coupled and counterbalancing roles of RPTKs and RPTPs are well defined in other systems. In this study, we evaluated the promoter activity and immunolocalization of two endothelial RPTPs, VE-PTP and PTPμ, in developing and adult renal vasculature using the heterozygous LacZ knock-in mice and specific antibodies. In adult kidneys, both VE-PTP and PTPμ were expressed in the endothelium of arterial, glomerular, and medullary vessels, while their expression was highly limited in peritubular capillaries and venous endothelium. VE-PTP and PTPμ promoter activity was also observed in medullary tubular segments in adult kidneys. In embryonic (E12.5, E13.5, E15.5, E17.5) and postnatal (P0, P3, P7) kidneys, these RPTPs were expressed in ingrowing renal arteries, developing glomerular microvasculature (as early as the S-shaped stage), and medullary vessels. Their expression became more evident as the vasculatures matured. Peritubular capillary expression of VE-PTP was also noted in embryonic and postnatal kidneys. Compared to VE-PTP, PTPμ immunoreactivity was relatively limited in embryonic and neonatal renal vasculature and evident immunoreactivity was observed from the P3 stage. These findings indicate 1) VE-PTP and PTPμ are expressed in endothelium of arterial, glomerular, and medullary renal vasculature, 2) their expression increases as renal vascular development proceeds, suggesting that these RPTPs play a role in maturation and maintenance of these vasculatures, and 3) peritubular capillary VE-PTP expression is down-regulated in adult kidneys, suggesting a role of VE-PTP in the development of peritubular capillaries.

## Introduction

Proper development of renal vasculature involves the coordination of several processes, including endothelial cell proliferation, migration, homotypic and heterotypic intercellular adhesion, and morphogenesis. In recent decades, a body of studies has defined the pivotal role of endothelial receptor tyrosine kinases (RPTKs) and their activating ligands in promoting and coordinating renal vascular development, especially in glomerular microvascular development [1, 2]. These include VEGFs and VEGF receptors, angiopoietins and Tie receptors, and ephrins and Eph receptors [2–4]. However, the expression and role of endothelial receptor-type tyrosine phosphatases (RPTPs) during the development of renal vasculature are poorly understood, though coupled and counterbalancing roles of RPTKs and RPTPs are well defined in neural systems [5].

Within a large family of RPTPs, Vascular endothelial-PTP (VE-PTP), PTPμ, and CD148 were shown to be expressed in vascular endothelium at high levels. VE-PTP is a R3 family RPTP, composed of an extracellular segment of fibronectin type-III repeats, a transmembrane domain, and a single intracellular PTP domain. In embryonic and postnatal tissues, VE-PTP is specifically expressed in endothelial cells [6, 7], and homozygous mice exhibit severe vascular remodelling defects, causing lethality at 10 days of gestation, although vasculogenesis occurs normally [6, 7]. VE-PTP was shown to be associated with two endothelial membrane proteins, angiopoietin receptor Tie-2 and VE-cadherin [8, 9], which both play essential roles in angiogenesis. VE-PTP dephosphorylates the Tie-2 receptor and negatively regulates angiopoietin-1 signalling [10, 11]. An anti-VEPTP polyclonal antibody that down regulates VE-PTP expression increases Tie-2 activity and promotes endothelial proliferation and vessel enlargement [10]. Furthermore, recent studies demonstrated that small-molecule VE-PTP inhibitors (AKB-9778, AKB-9785) increases Tie-2 activity, stabilizes vessels, and inhibits neovascularization in mouse models of neovascular age-related macular degeneration and ischemic retinal neovascularization [11] and reduces infarct sizes and vessel permeability in experimental stroke [12]. Interestingly, a more recent study has shown that VE-PTP inhibition stabilizes endothelial junctions via Tie-2 even in the absence of VE-cadherin [13]. These findings indicate an important role for VE-PTP to balance Tie2 activity and regulate vessel formation and stability. On the other hand, VE-PTP was also shown to interact with VE-cadherin through its extracellular segment, dephosphorylate VE-cadherin and γ-catenin, and enhance the adhesive function of VE-cadherin [9, 14, 15], indicating a role of VE-PTP in endothelial barrier function. To date, the extracellular ligands for VE-PTP remain unknown.

PTPμ is a R2B family RPTP, which is comprised of a MAM (Meprin, Xenopus A5, MU)-domain containing ectodomain, a transmembrane domain, and an intracellular segment possessing a tandem arrangement of PTP domains [16]. PTPμ is abundantly expressed in both developing and mature arterial endothelial cells in various organs, including heart, lung, liver, and aorta [17, 18]. The ectodomain of PTPμ interacts homophilically and mediates endothelial cell-cell adhesion [18–20]. Furthermore, PTPμ associates with and dephosphorylates VE-cadherin and enhances endothelial cell-cell adhesion and barrier function [21–23].

Collectively, these findings demonstrate important roles of endothelial RPTPs in vessel formation and function. However, the expression and role of these RPTPs in renal vasculature are poorly understood. Here we evaluated the expression of VE-PTP and PTPμ in renal vascular development using heterozygous LacZ knock-in mice and specific antibodies. Our results demonstrate that; 1) both VE-PTP and PTPμ are expressed in the endothelium of arterial, glomerular, and medullary vasculatures in developing and adult kidneys; 2) their expression increases as renal vascular development proceeds; 3) the expression of these RPTPs is highly limited in venous endothelium. Furthermore, our data also demonstrate that VE-PTP is expressed in peri-tubular capillary vessels in embryonic and postnatal mouse kidneys and is down regulated in adult kidneys. These findings suggest a site and time specific role of these RPTPs in the formation and maintenance of renal vasculature.

## Materials and methods

### Animals

The promoter activity of VE-PTP or PTPμ was assessed using the VE-PTP^tlacZ^/+ [7] and PTPμ^tlacZ^/+ [17] heterozygous mice in which the β-galactosidase (LacZ) reporter gene is expressed under the control of the endogenous VE-PTP or PTPμ promoter, respectively. Wild-type mice were used for immunohistochemical study. Since aged mice show glomerular immunoglobulin depositions, 4–6 week-old mice were used for immunostaining.

### Ethics statement

All animal procedures were approved by the Vanderbilt University Institution of Animal Care and Use Committee and conducted in accordance with institutional guidelines. Mice were euthanized by inhalation of carbon dioxide overdose and subsequent cervical dislocation. For embryos, the uterus was removed and dissected, and the embryos were euthanized by head amputation with a sharp scissors or blade.

### β-galactosidase histochemistry

Kidneys were sampled from the embryos or mice at the indicated developmental stage. β-galactosidase histochemistry was performed as described previously [24]. In brief, the dissected kidney tissues were washed in PBS and fixed with 4% paraformaldehyde in PBS for 5–15 min with PTPμ^tlacZ^/+ kidneys and 15–30 min with VE-PTP^tlacZ^/+ kidneys, respectively. Kidney tissues were rinsed twice in cold PBS and permeabilized with PBS containing 0.02% NP-40, 0.01% sodium deoxycholate and 2 mM MgCl_2_ for 30 min at 4°C. Color development was carried out in PBS solution containing 0.02% NP-40, 5 mM potassium ferricyanide, 5 mM potassium ferrocyanide, 2 mM MgCl_2_, and 1 mg/ml 5-bromo-4-chloro-3-indolyl-D-galactopyranoside (X-gal; Sigma, St. Louis, MO) overnight at RT. Tissues were post-fixed with 4% paraformaldehyde in PBS for 60 min at 4°C, washed in PBS, dehydrated through a graded ethanol series and Histo-Clear (National Diagnostics, Atlanta, GA), and then embedded in paraffin. Sections (5 μm thick) were collected, dehydrated, mounted, and photographed by light microscopy (Zeiss Axioskop 40; Carl Zeiss, Thornwood, NY).

Immunohistochemistry for CD31 was superimposed on β-galactosidase histochemisty as follows. After β-galactosidase histochemistry, kidney sections were dewaxed, dehydrated, and blocked with 0.1% FBS/PBS for 30 min at RT. The antigen retrieval was carried out using a microwave method according to the manufacture’s protocol (HistoBioTec, Miami Beach, FL). Then the sections were incubated with anti-CD31 rat monoclonal (1:200, clone SZ31; HistoBioTec) overnight at 4°C. Sections were washed in cold PBS and immunoreactions were detected using VECTASTATIN Rat IgG ABC Kit (Vector Laboratories, Burlingame, CA) and visualized using the Peroxidase DAB Substrate Kit (Vector Laboratories). Dehydrated tissue sections were mounted (Cytoseal XYL; Thermo Fisher Scientific Inc., Waltham, MA) and photographed by light microscopy (Zeiss Axioskop 40).

### Immunofluorescence and immunohistochemistry procedures

Kidney tissues of wild-type mice were placed in optimal cutting temperature (OCT) compound (Sakura Finetek U.S.A. Inc. Torrance, CA) and snap-frozen in a dry ice-acetone bath. Cryostat sections (4 μm) were fixed in 100% acetone for 10 min at −20°C, washed three times in cold PBS, and blocked with 5% normal goat serum for 20 min at RT. For PTPμ immunofluorescence staining, the Fab fragments (10 μg/ml) of unconjugated secondary antibody, goat anti-mouse IgG antibody (Jackson ImmunoResearch Laboratories, West Grove, PA), were added to 5% normal goat serum to block the secondary antibody’s reaction to endogenous mouse IgG. After blocking, the sections were incubated with the following primary antibodies overnight at 4°C; anti-VEPTP rat monoclonal antibody (Clone 109.3, 10 μg/ml, provided by Dietmar Vestweber, Max Planck Institute) [6], anti-VEPTP rabbit polyclonal antibody (affinity purified) raised against the extracellular fibronectin type III-like domains 1–8 (1 μg/ml), or anti-PTPμ mouse monoclonal antibody (Clone SBK15, 1:200 dilution, Millipore, Temecula, CA) [18]. These primary antibodies have been successfully applied to rodent tissue immunohistochemistry [6, 18]. The specificity of the anti-VEPTP rabbit polyclonal antibody was validated by immunoblotting of VE-PTP transfected and knocked-down cells. The sections were then washed in cold PBS and incubated with the secondary antibodies, Alexa Fluor 546-conjugated goat anti-rat IgG or Alexa Fluor 546-conjugated goat anti-mouse IgG antibodies (2 μg/ml; both were from Invitrogen, Oregon, IL) for 30min at RT. Washed sections were mounted (Fluorogel with Tris buffer; Electron Microscopy Sciences, Hatfield, PA) and photographed by confocal microscopy (Zeiss LSM510). In some studies, immunoreactions to VE-PTP were detected and visualized using the biotinylated secondary antibody and VECTASTAIN ABC System (Vector Laboratories). In brief, cryostat sections were fixed as described above and endogenous biotin was blocked using the Avidin/Biotin Blocking Kit (Vector Laboratories) according to the manufacturer’s instruction. Sections were then blocked with 5% normal rabbit serum for 20 min at RT and then incubated with anti-VE-PTP rat monoclonal antibody for 60 min at RT. Sections were washed in cold PBS and immunoreactions were detected using VECTASTATIN Rat IgG ABC Kit (Vector Laboratories) and visualized using the Peroxidase DAB Substrate Kit (Vector Laboratories). Dehydrated tissue sections were mounted (Cytoseal XYL; Thermo Fisher Scientific Inc.) and photographed by light microscopy (Zeiss Axioskop 40).

For the double-labeling immunofluorescence study, cryostat sections were blocked and incubated with anti-VE-PTP rat monoclonal antibody or anti-VE-PTP rabbit polyclonal antibody overnight at 4°C and subsequently incubated with the secondary antibody (Alexa Fluor 546-conjugated goat anti-rat IgG or Alexa Fluor 546-conjugated goat anti-rabbit IgG, Invitrogen). After washing in cold PBS, the sections were incubated with fluorescein isothiocyanate (FITC)-conjugated anti-mouse CD31 rat monoclonal antibody (MEC13.3) (5 μg/ml; BD Biosciences, San Jose, CA) for 60 min at RT, washed, mounted, and photographed by confocal microscopy (Zeiss LSM510). The double-immunolabeling staining of PTPμ and CD31 was carried out as follows. Cryostat sections were blocked and incubated with anti-PTPμ mouse monoclonal antibody (SBK15, Millipore) and anti-mouse CD31 rat monoclonal antibody (MEC13.3, BD Biosciences) overnight at 4°C. Subsequently, the sections were incubated with the secondary antibodies (FITC-conjugated goat anti-mouse IgG, Rhodamine-conjugated goat anti-rat IgG, Jackson Immunoresearch) for 30 min at RT. After washing in cold PBS, the sections were mounted and photographed by confocal microscopy (Zeiss LSM510).

## Results

### Promoter activity of VE-PTP and PTPμ in adult renal vasculature

**Fig 1:**
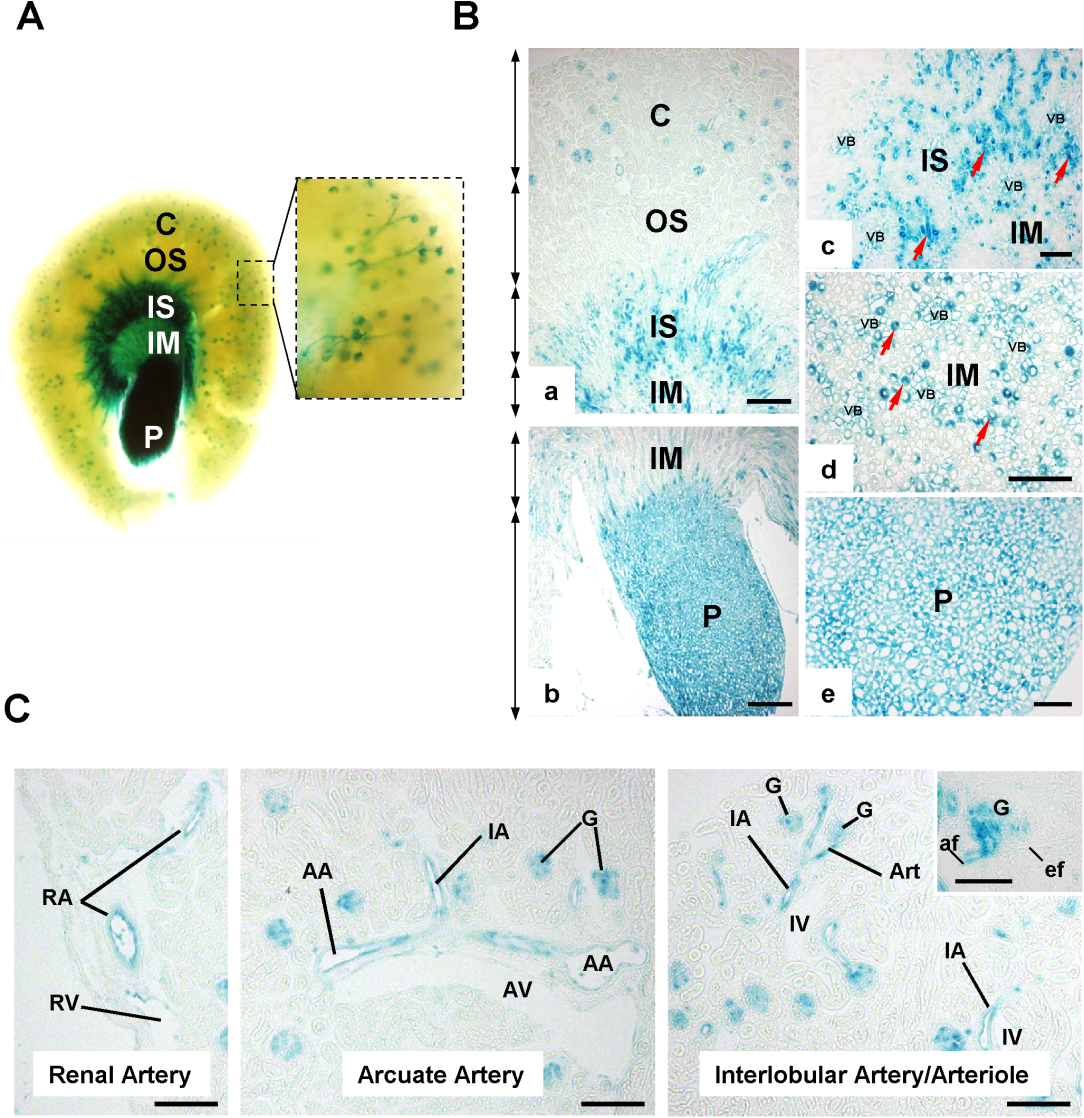
VE-PTP promoter activity in adult mouse kidney. **(A)** Whole mount X-gal staining of adult VE-PTP^tlacZ/+^ mouse kidney. Evident β-galactosidase activity is observed in renal cortex (C), inner stripe (IS) of outer medulla, inner medulla (IM), and papilla (P). Right panel shows vascular distribution of β-galactosidase activity in renal cortex. C, cortex; OS, outer stripe; IS, inner stripe; IM, inner medulla; P, papilla. **(B)** β-galactosidase activity in adult VE-PTP^tlacZ/+^ mouse kidney sections. In medulla, β-galactosidase activity is observed in vascular bundle (VB), subpopulations of medullary tubules (red arrows in panels c and d), and papillary cells (panel e). VB, vascular bundle. **(C)** β-galactosidase activity in cortical renal vasculature. RA, renal artery: RV, renal vein; AA, arcuate artery; AV, arcuate vein; IA, interlobular artery; IV, interlobular vein; Art, arteriole; G, glomerulus; af, afferent arteriole; ef, efferent arteriole. Note: VE-PTP promoter activity is limited in efferent arterioles, peritubular capillaries, and venous circulations. Scale bar, 200 μm in B-a and B-b; 100 μm in B-c, B-d, B-e, and C; 50 μm in an insert of C.

The promoter activity of VE-PTP and PTPμ were assessed using the VE-PTP^tlacZ^/+ and PTPμ^tlacZ^/+ heterozygous mice. Both mice strains were healthy with morphologically normal kidneys. In adult VE-PTP^tlacZ^/+ kidneys (4–6 weeks of age), evident VE-PTP promoter activity was observed in renal cortex, inner stripe of outer medulla (ISOM), inner medulla (IM), and papilla (P) (**Fig 1A and 1B**). In cortex, VE-PTP promoter activity was observed in arterial vasculature and glomerulus, while it was limited in post-glomerular circulation including efferent arterioles, peritubular capillaries, and veins (**Fig 1C**). In medulla, VE-PTP promoter activity was observed in medullary vessels in ISOM, IM, and P (**Fig 1B**). Interestingly, VE-PTP transcription was also observed in medullary tubular segments, yet endothelial-specific expression of VE-PTP was shown in previous reports [6–8]. These include segments of Henle’s loop and collecting ducts (**Fig 1B**, **S1A Fig**). Wild-type kidneys were negative for β-galactosidase activity.

**Fig 2:**
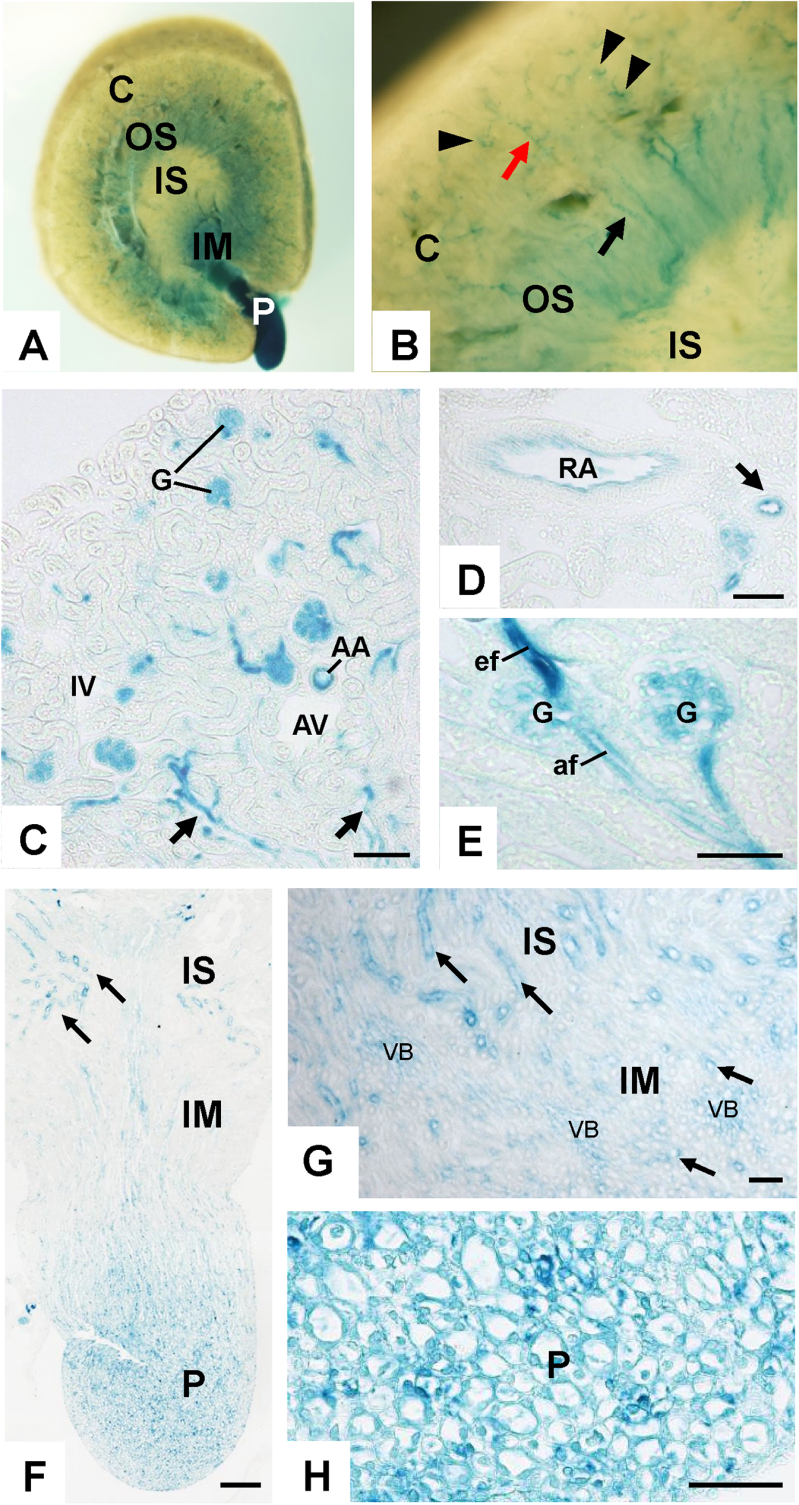
PTPμ promoter activity in adult mouse kidney. **(A and B)** Whole mount X-gal staining of adult PTPμ^tlacZ/+^ mouse kidney. Evident β-galactosidase activity is observed in papilla (P). Panel B shows a magnified view showing vascular distribution of β-galactosidase activity in renal cortex. Arrowheads indicate glomeruli, a red arrow shows interlobular artery, and a black arrow shows vasa recta. C, cortex; OS, outer stripe; IS, inner stripe; IM, inner medulla; P, papilla. **(C)** β-galactosidase activity in renal cortex of adult PTPμ^tlacZ/+^ mouse. β-galactosidase activity is observed in arterial and glomerular circulations and vasa recta (arrows), while it is limited in peritubular capillaries and venous circulations. AA, arcuate artery; AV, arcuate vein; IA, interlobular artery; IV, interlobular vein; G, glomerulus. **(D)** β-galactosidase activity in renal artery and its branch artery (arrow). **(E)** β-galactosidase activity in glomerular microcirculation. Strong PTPμ promoter activity is observed in efferent limb. G, glomerulus; af, afferent arteriole; ef, efferent arteriole. **(F through H)** β-galactosidase activity in renal medulla. β-galactosidase activity is observed in tubular segments (arrows) in inner stripe (IS) of outer medulla, inner medulla (IM), and papilla (P) as well as in vascular components in inner medulla (VB in G) and papilla. Scale bar, 100 μm in C; 200 μm in F; 50 μm in D, E, G, and H.

In adult PTPμ^tlacZ^/+ kidneys (4–6 weeks of age), PTPμ promoter activity was observed in arterial vasculature, glomerulus, and medullary vessels, while it was limited in peritubular capillaries and venous circulations (**Fig 2**). Strong PTPμ promoter activity was observed in the efferent arteriole of glomerulus (**Fig 2E**). In the medulla, PTPμ promoter activity was also observed in subpopulations of medullary tubules (**Fig 2F through 2H**). Superimposed immunohistochemistry for Tamm-Horsfall protein (THP) or histochemistry of *Dolichos biflorus agglutin* (DBA) lectin showed that PTPμ promoter activity is present in collecting ducts, but not in Henle’s thick ascending limbs (**S1B Fig**). Although β-galactosidase activity was observed in tubules in the outer stripe of outer medulla (OSOM) (**Fig 2A and 2B**), we characterized this as non-specific activity, as similar activity was observed in wild-type kidneys (**S2 Fig**). Since PTPμ promoter activity was weaker than that of VE-PTP, we applied milder fixation to the β-galactosidase histochemistry of PTPμ^tlacZ^/+ kidneys, which explains why non-specific β-galactosidase activity was observed in OSOM in PTPμ^tlacZ^/+, but not VE-PTP^tlacZ^/+, kidneys.

**Fig 3:**
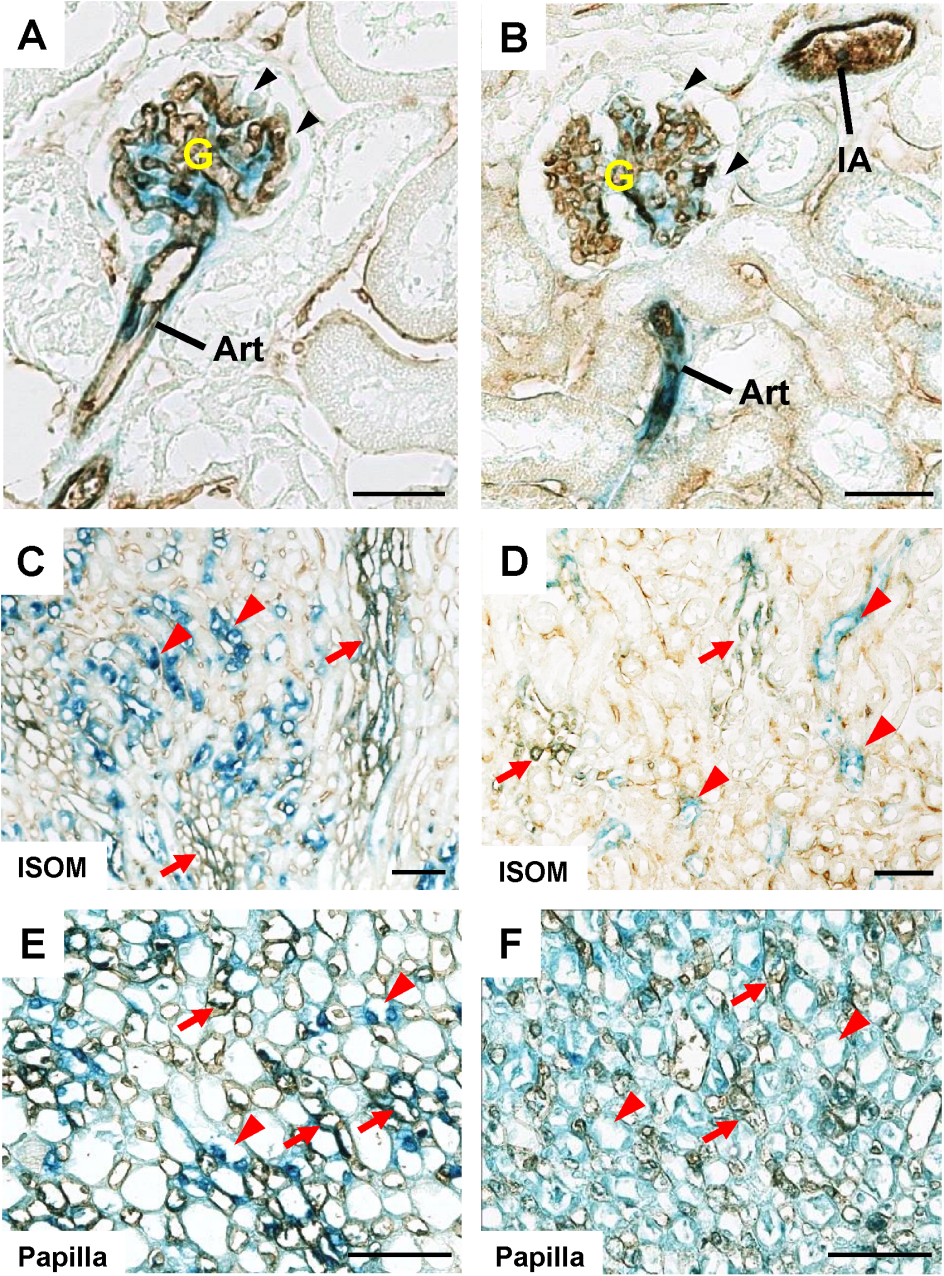
Anti-CD31 immunohistochemistry combined with β-galactosidase histochemistry of adult VE-PTP^tlacZ/+^ and PTPμ^tlacZ/+^ mice kidneys. **(A through F)** Immunohistochemistry for CD31 was superimposed on β-galactosidase histochemistry of adult VE-PTP^tlacZ/+^ (A, C, E) and PTPμ^tlacZ/+^ (B, D, F) mice kidneys. CD31 is co-localized with β-galactosidase activity in glomeruli and arterioles (panels A and B) and medullary vessels (red arrows) in inner stripe of outer medulla (ISOM) and papilla (panels C through F) in VE-PTP^tlacZ/+^ and PTPμ^tlacZ/+^ mice kidneys. Glom, glomerulus; Art, arteriole; IA, interlobular artery. Notes: β-galactosidase activity is absent in podocytes (black arrows in A and B). β-galactosidase activity is also observed in tubular segments (red arrowheads) that are not labelled with CD31. Scale bar, 25 μm in A and B; 50 μm in C through F.

The endothelial distribution of VE-PTP and PTPμ promoter activity was assessed by superimposing anti-CD31 immunohistochemistry on β-galactosidase histochemistry (**Fig 3**). As shown in this Fig, CD31 immunostain overlapped with β-galactosidase activity in arterial and glomerular vasculature (**Fig 3A and 3B**) and in medullary vessels (red arrows, **Fig 3C through 3F**) in VE-PTP^tlacZ^/+ and PTPμ^tlacZ^/+ kidneys. The VE-PTP and PTPμ promoter activity in medullary tubular cells were also indicated by the negative stain of CD31 in these cells (red arrowheads, **Fig 3C through 3F**).

### Promoter activity of VE-PTP and PTPμ in developing renal vasculature

The promoter activity of VE-PTP and PTPμ were assessed in developmental kidneys at embryonic and postnatal stages using the VE-PTP^tlacZ^/+ and PTPμ^tlacZ^/+ mice and β-galactosidase histochemistry. In mice, metanephrogenesis begins at embryonic days 10 and 11 (E10-E11) when the ureteric buds (UBs) that branch from the Wolffian duct migrate into the metanephric mesenchyme [25]. The invasion of UBs induces aggregation of mesenchyme around its advancing edge. By E12, UBs branch a few times and capillary networks appear between metanephric epithelia. Mesenchymal aggregates form glomerular and tubular components and UBs generate collecting ducts. Renal vasculature is thought to be formed by both angiogenesis and vasculogenesis with endothelial cells of extra-renal and renal origin [26, 27]. The first metanephric glomeruli are formed by E14 and new nephron layers are developed by postnatal day 1 (P1).

**Fig 4:**
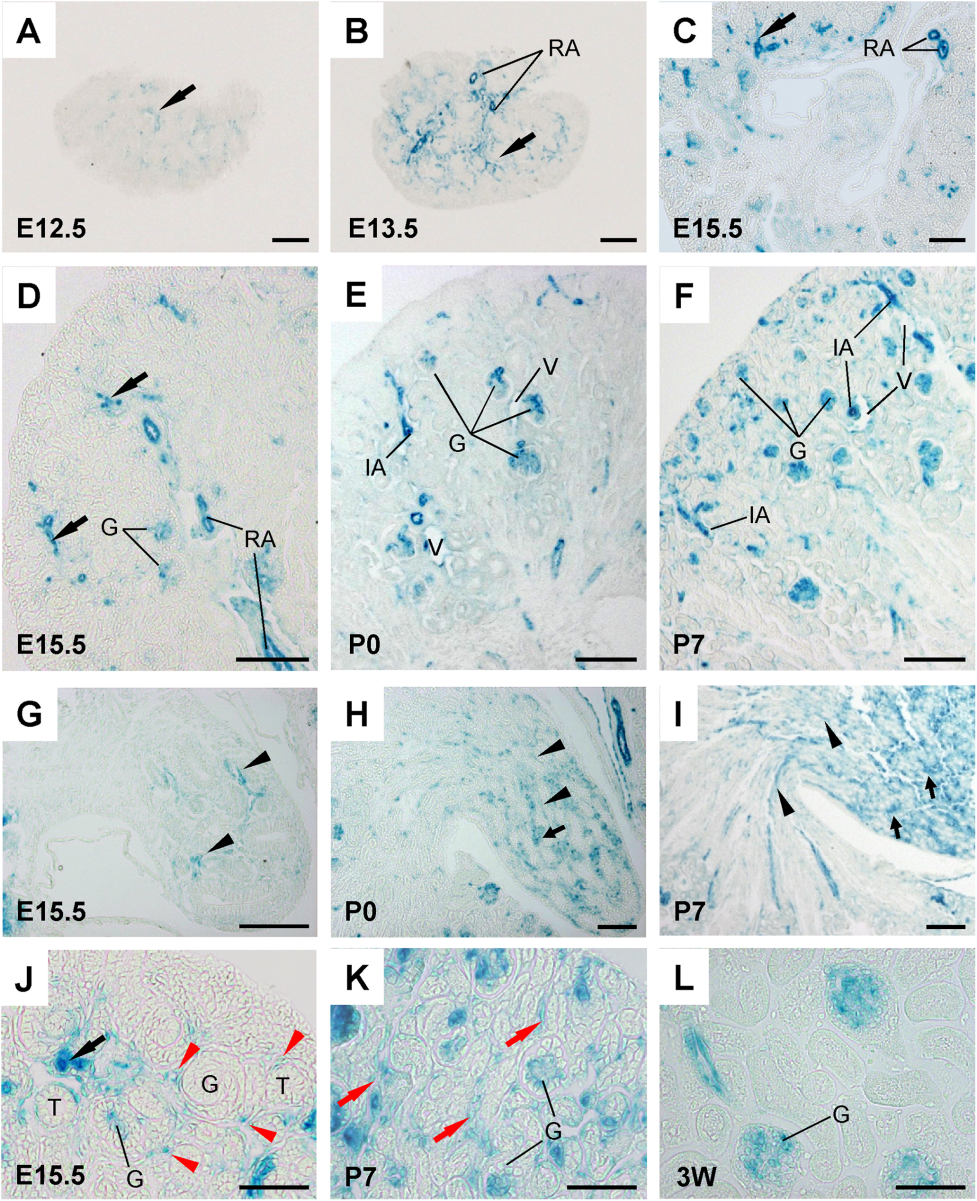
VE-PTP promoter activity in developing mouse kidneys. **(A through L)** β-galactosidase histochemistry of developing VE-PTP^tlacZ/+^ mouse kidneys. In embryonic kidneys, evident VE-PTP activity is observed in penetrating and ingrowing renal arteries (black arrows in panels A, B, C, D, and J) and maturing glomeruli (G) (panels D and J). VE-PTP promoter activity is also observed in developing medullar vessels (arrowheads in panel G). In postnatal kidneys, VE-PTP promoter activity is observed in renal arterial vessels (panels E and F), maturing glomeruli (G) (panels E, F, and K), and medullary vessels (arrow heads in panels H and I) and tubular subpopulations (arrows in panels H and I). Note: VE-PTP promoter activity is observed in the cells intermittently distributed around developing glomeruli and tubules (red arrowheads in panel J) and in peri-tubular capillaries in postnatal kidneys (red arrows in panel K). The peritubular capillary VE-PTP promoter activity is down-regulated in 3-week old mouse kidney (panel L). RA, renal artery; G, glomerulus; IA, interlobular artery; V, venous vessel; T, developing tubules. Scale bar, 100 μm in A through I; 50 μm in J through L.

At early nephrogenesis (E12.5-E13.5), VE-PTP promoter activity was observed in renal arterial vessels that are penetrating and ingrowing in metanephros (**Fig 4A and 4B, S3 Fig**). VE-PTP promoter activity was also noted in the juxta-medullary glomeruli and in the cells that are distributed around arterial vessels and mesenchymal condensates (**S3 Fig**). In E15.5–E17.5 kidneys, VE-PTP promoter activity was observed in ingrowing renal arterial vessels and glomeruli (**Fig 4C, 4D and 4J**), and in the cells dispersively distributed around the developing glomeruli and tubules (red arrows, **Fig 4J**), and medullary vessels (**Fig 4G**). At postnatal stages (P0, P3, and P7), VE-PTP promoter activity was observed in renal arterial vessels (**Fig 4E and 4F**), developing glomeruli (**Fig 4E, 4F and 4K**), peritubular capillaries (**Fig 4K**), and medullary vessels and tubular subpopulations (**Fig 4H and 4I, S4 Fig**). Similar to the adult kidneys, VE-PTP promoter activity was limited in venous vessels in developing kidneys (**Fig 4E and 4F**). Interestingly, we also noted that VE-PTP promoter activity is down-regulated in peritubular capillaries in 3 week-old kidneys (**Fig 4L**).

**Fig 5:**
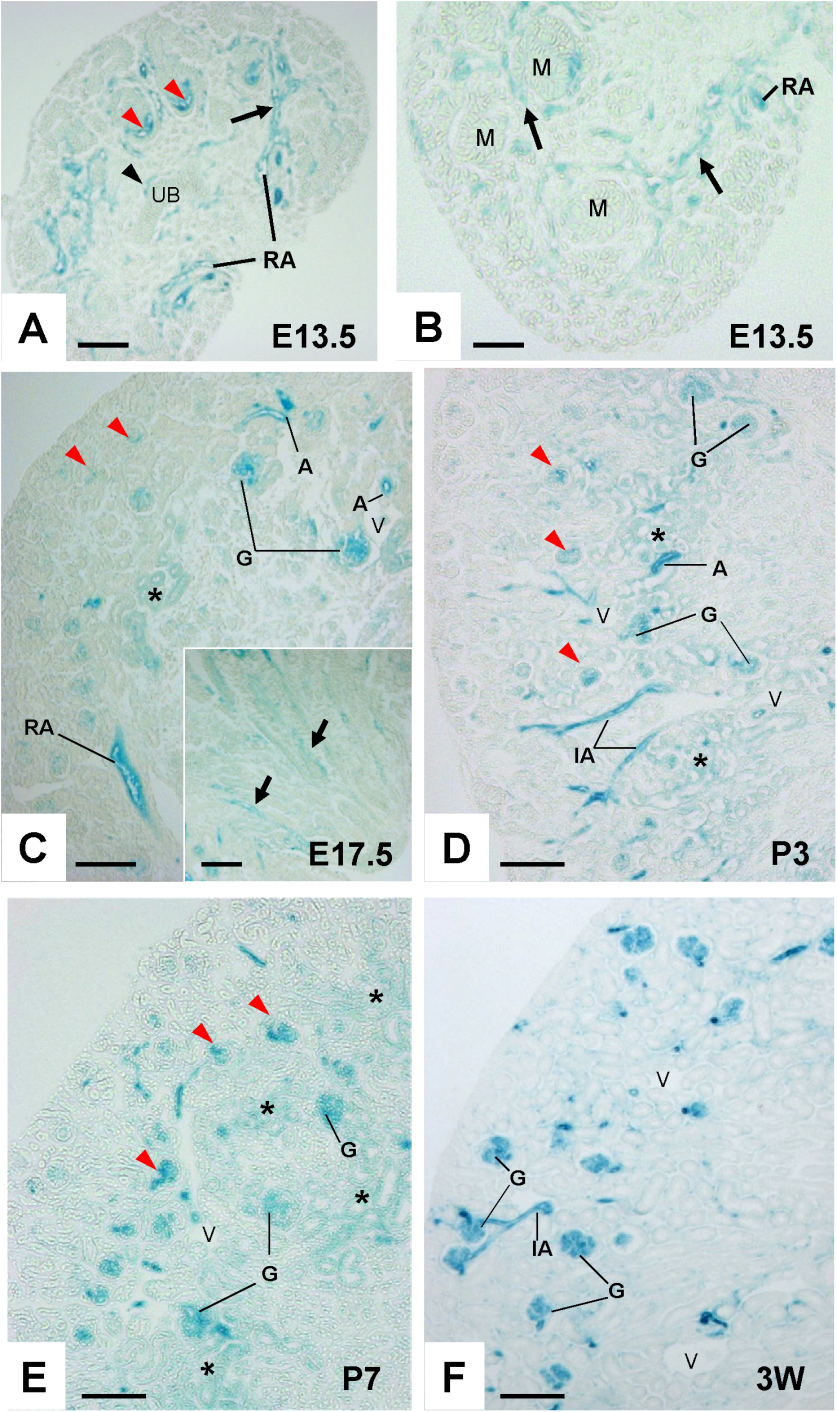
PTPμ promoter activity in developing mouse kidney. **(A and B)** β-galactosidase activity in E13.5 kidneys. β-galactosidase activity is observed in ingrowing renal arteries (black arrows), vessels (a black arrowhead) adjacent to ureteric bud (UB), and vascular clefts (red arrowheads) in developing juxta-medullary glomeruli. RA, renal artery; M, mesenchymal condensate. **(C through E)** β-galactosidase activity in developing PTPμ^tlacZ/+^ mouse kidneys at the stages of E17.5 (C), P3 (D), and P7 (E). Evident β-galactosidase activity is observed in arterial vessels (A) and juxta-medullary maturing glomeruli (G). β-galactosidase activity is also observed in developing cortical glomeruli (red arrowheads in panel C) and its degree increases as the kidney development proceeds (red arrowheads in panels D and E). β-galactosidase activity is also noted in developing medullar vessels (arrows in an insert of panel C). Asterisks show non-specific β-galactosidase activity in tubules at cortico-medullary junction. RA, renal artery; A, arterial vessel; IA, interlobular artery; G, glomerulus. **(F)** β-galactosidase activity in renal cortex of 3-week old PTPμ^tlacZ/+^ mouse. Evident β- galactosidase activity is observed in arterial and glomerular, but not venous, circulations. G, glomerulus; IA, interlobular artery; V, vein. Scale bar, 100 μm in A, C, D, E, and F; 50 μm in B.

Similar to VE-PTP, PTPμ promoter activity was observed in ingrowing renal arterial vessels in E13.5 embryonic kidneys (**Fig 5A and 5B**). PTPμ promoter activity was also observed in vascular clefts in developing juxta-medullary glomeruli and the vessels adjacent to ureteric buds (**Fig 5A**). At the stages of E17.5, P3 and P7, PTPμ promoter activity was observed in arterial vessels and developing glomeruli (**Fig 5C, 5D and 5E**). The juxta-medullary glomeruli showed stronger PTPμ promoter activity compared with cortical glomeruli, and PTP promoter activity in cortical glomeruli increased as the kidney development proceeded (red arrowheads, **Fig 5C through 5F**). β-galactosidase activity was also noted in developing medullar vessels (an insert, **Fig 5C**) and tubular segments in outer medulla in postnatal kidneys (data not shown). PTPμ promoter activity was limited in peritubular capillaries, venous vessels, and tubules in the inner medulla and papilla in developing kidneys.

**Fig 6:**
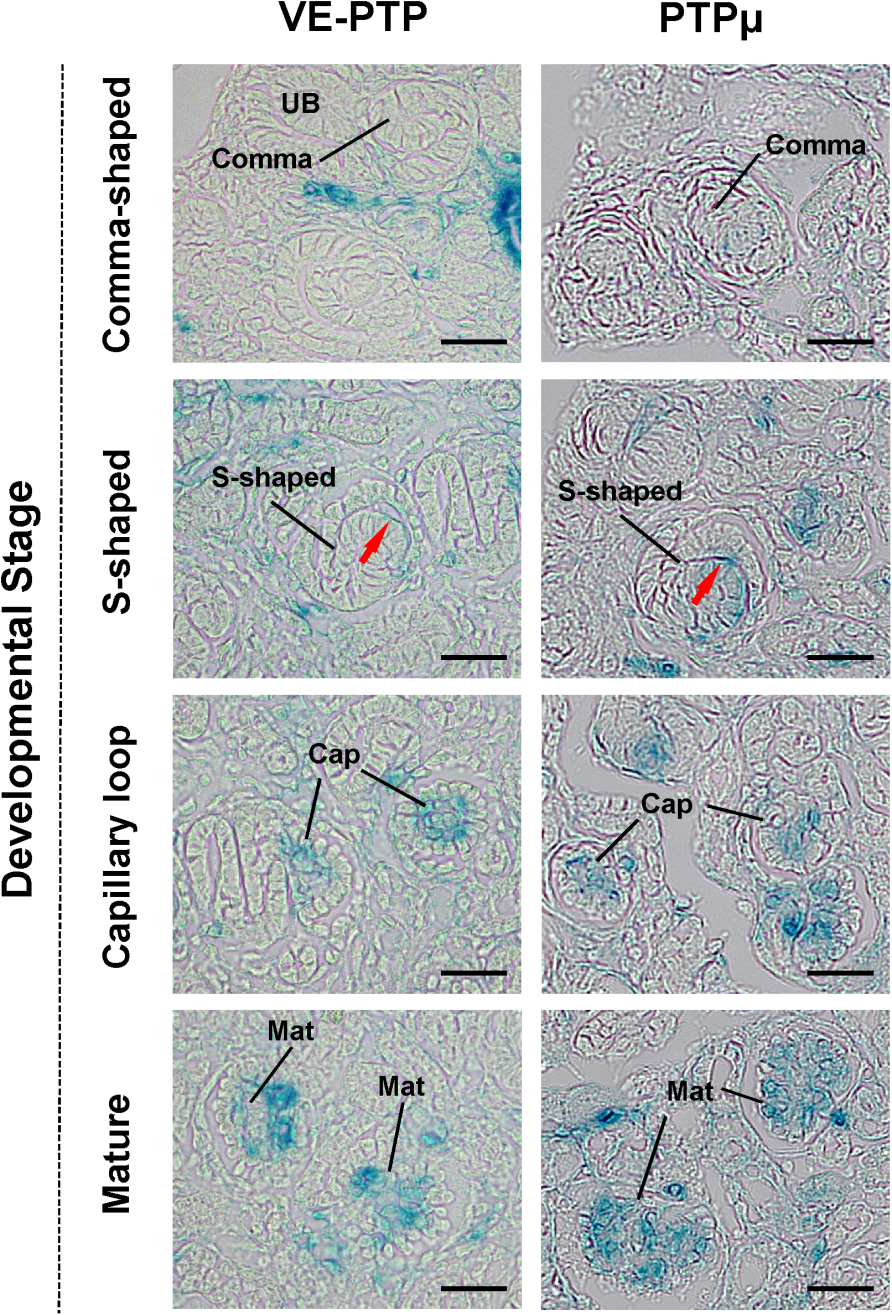
VE-PTP and PTPμ promoter activity in developing mouse glomeruli. Representative β-galactosidase activity in developing VE-PTP^tlacZ/+^ (left panels) and PTPμ^tlacZ/+^ (right panels) mice glomeruli is shown. The promoter activities of VE-PTP and PTPμ are observed as early as in S-shaped glomeruli, and it increases as the glomeruli mature. Red arrows indicate vascular clefts of S-shaped glomeruli. Cap, capillary loop stage glomeruli; Mat, maturing glomeruli. Scale bar, 25 μm.

The **Fig 6**shows VE-PTP and PTPμ promoter activity in developing cortical glomeruli. VE-PTP and PTPμ promoter activity was observed in vascular clefts (red arrows) of S-shaped glomeruli, and the promoter activity of these RPTPs became more evident as glomerular development proceeded. The transcription of VE-PTP and PTPμ was limited to intraglomerular space, and podocytes were negative for the expression of these RPTPs. The promoter activity of these RPTPs was not observed in comma-shaped glomeruli.

**Fig 7:**
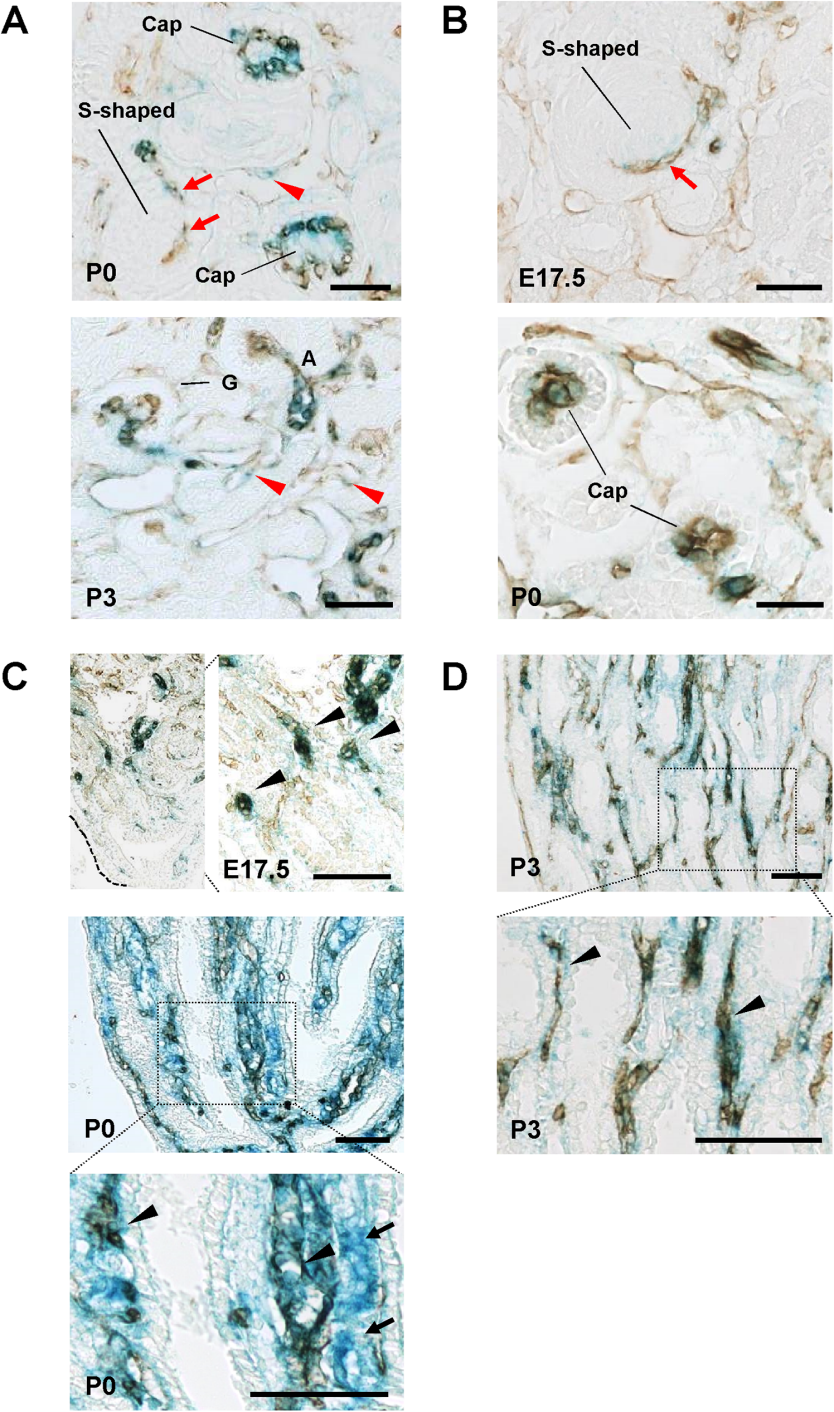
Anti-CD31 immunohistochemistry combined with β-galactosidase histochemistry of developing VE-PTP^tlacZ/+^ and PTPμ^tlacZ/+^ mice kidneys. **(A through D)** Immunohistochemistry for CD31 was superimposed on β-galactosidase histochemistry in developing VE-PTP^tlacZ/+^ (A, C) and PTPμ^tlacZ/+^ (B, D) mice kidneys. Panels A and B show cortical area. Panels C and D display medullary region. In both mice, CD31 and β-galactosidase activity are colocalized in developing glomerular capillaries, including vascular clefts of S-shaped glomeruli (red arrows), and developing medullary vessels (black arrowheads in panels C and D). In VE-PTP^tlacZ/+^ kidneys, β-galactosidase activity is also observed in endothelial cells forming peritubular capillaries (red arrowheads in panel A). Notes: β-galactosidase activity is observed in medullary tubular segments (black arrows) that are not labelled with CD31 in P0-stage VE-PTP^tlacZ/+^ mouse kidney. Scale bar, 25 μm in A, C; 50 μm in B, D.

The endothelial distribution of VE-PTP and PTPμ promoter activity was assessed in developing kidneys by superimposing anti-CD31 immunohistochemistry on β-galactosidase histochemistry (**Fig 7**). As shown in this Fig, CD31 immunostain overlapped well with β-galactosidase activity in developing arterial and glomerular vasculature (**Fig 7A and 7B**) and medullary vessels (**Fig 7C and 7D**) in VE-PTP^tlacZ^/+ and PTPμ^tlacZ^/+ kidneys. In developing VE-PTP^tlacZ^/+ kidneys, β-galactosidase activity was also observed in peritubular capillaries labelled with CD31 (red arrowheads, **Fig 7A**) and medullary tubular cells indicated by the negative stain of CD31 (arrows, **Fig 7C**).

### Immunolocalization of VE-PTP and PTPμ in developing and adult renal vasculature

**Fig 8:**
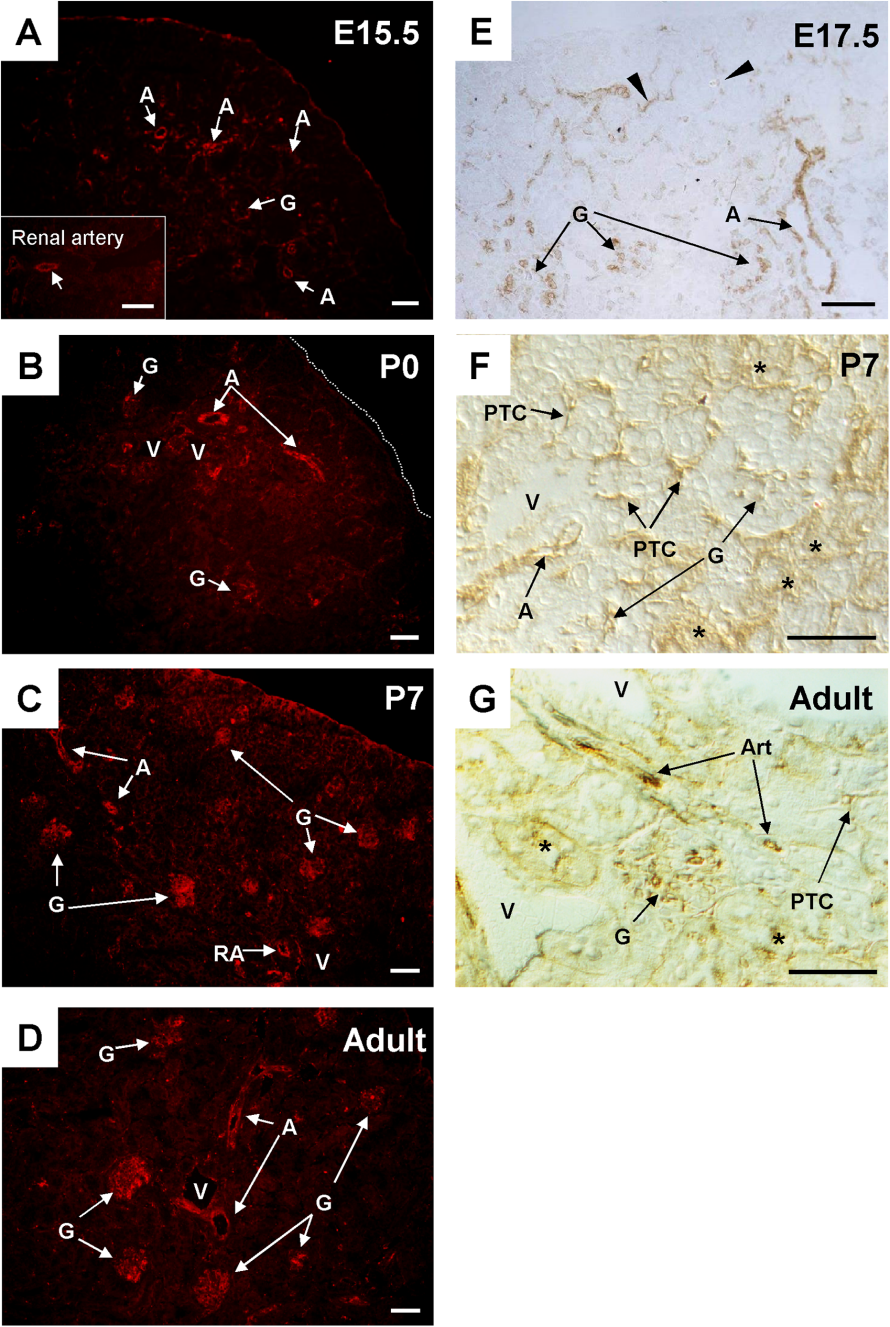
VE-PTP expression in developing and adult mouse renal vasculature. **(A through G)** Immunolocalization of VE-PTP was assessed by immunofluorescence staining (A though D) or immunohistochemistry with ABC method (E through G) as described in the “Materials & Methods”. At E15.5 (panel A) and E17.5 (panel E), VE-PTP expression is observed in ingrowing renal arteries (A), glomeruli (G) in juxta-medullary region, and the cells (arrows in panel E) surrounding developing glomeruli and tubules. At postnatal day 0 (panel B) and day 7 (panels C and F), VE-PTP is expressed in renal arterial vessels (A), maturing glomeruli (G), and peritubular capillaries (PTC). In adult kidney (panels D and G), VE-PTP is expressed in renal arterial vasculature and glomeruli, while its expression is limited in peritubular capillaries (PTC) and venous circulation (V). Asterisks in panels F and G indicate non-specific tubular staining. RA, renal artery; A, arterial vessel; Art, arteriole; G, glomerulus; PTC, peritubular capillary; V, vein. Scale bar, 50 μm in A, B, C, D, F, and G; 40 μm in E.

**Fig 9:**
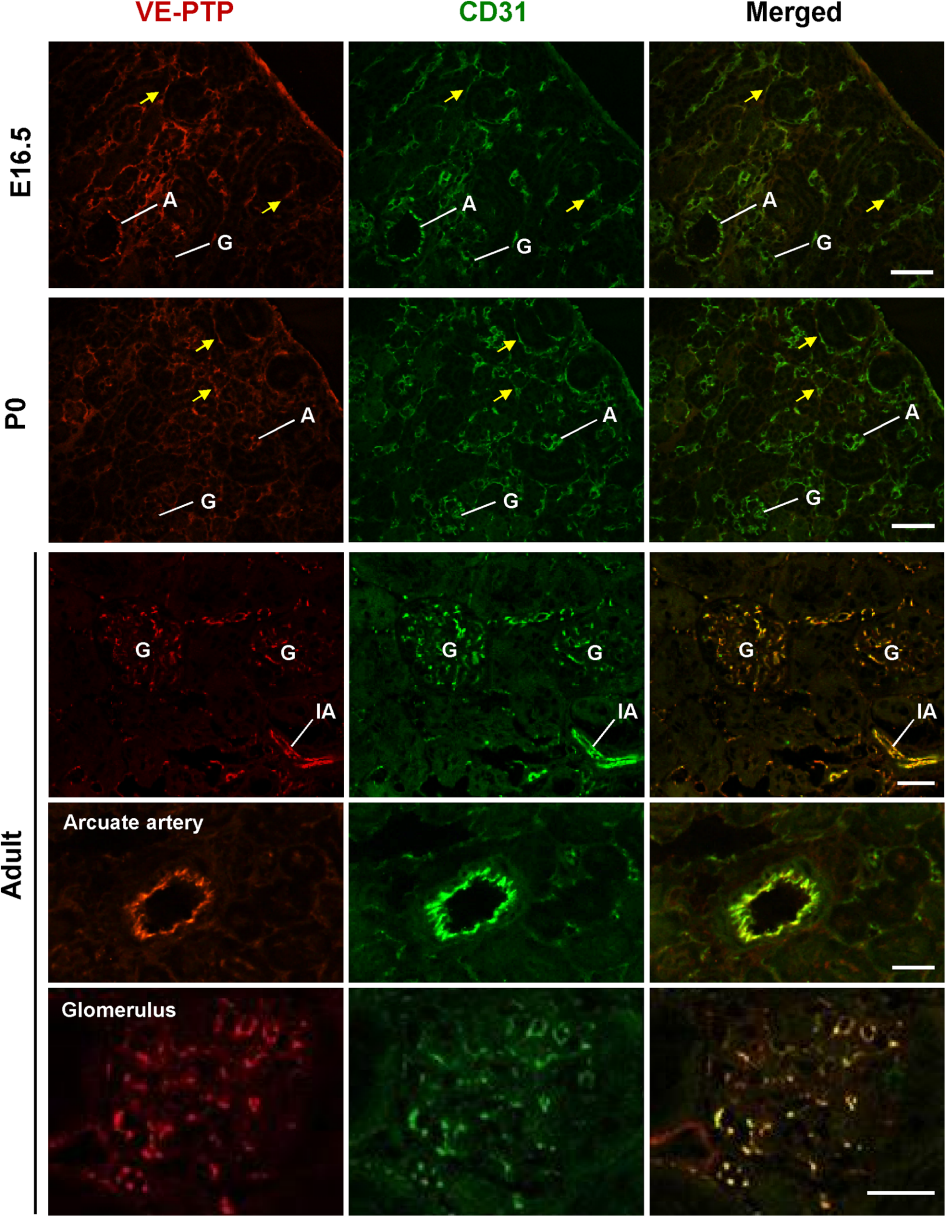
Co-immunostaining of VE-PTP and CD31 in developing and adult mice kidneys. Kidney sections from E16.5, P0 and adult mice were double immunolabeled for VE-PTP (red) and CD31 (green) as described in the “Materials & Methods”. In developing kidneys (E16.5 and P0), VE-PTP is expressed in endothelial cells in ingrowing arteries (A) and developing glomeruli (G), which are labeled with CD31. VE-PTP is also expressed in endothelial cells (yellow arrows) that distribute around the developing nephrons. In adult kidney, VE-PTP is expressed in endothelial cells in arterial and glomerular vasculature, while its expression is limited in peritubular capillaries. A, arterial vessel; IA, interlobular artery; G, glomerulus. Scale bar, 50 μm in E16.5 and P0 kidneys; 25 μm in adult kidney.

**Fig 10:**
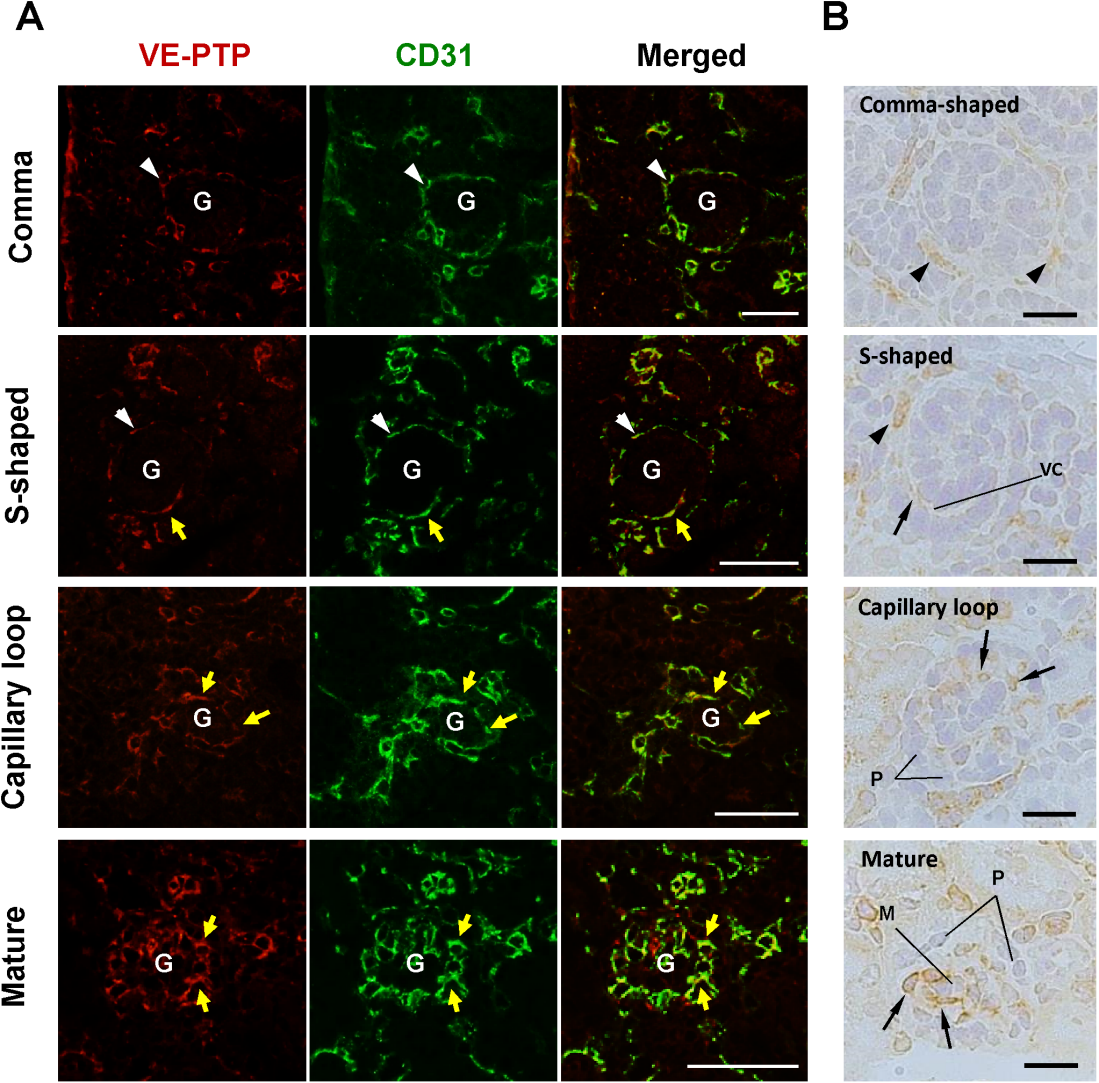
Co-immunostaining of VE-PTP and CD31 in developing mouse glomeruli. **(A)** Kidney sections from E16.5 and P0 mice were double immunolabeled for VE-PTP (red) and CD31 (green). **(B)** VE-PTP expression in developing glomeruli was also examined by immunohistochemistry with ABC method as described in the “Materials & Methods”. VE-PTP is expressed in glomerular endothelial cells (yellow arrows in panel A, black arrows in panel B) that are distributed in vascular clefts of S-shaped glomeruli and within capillary loop stage and maturing glomeruli. VE-PTP immunoreactivity is increased as glomerular capillary development proceeds. No VE-PTP immunoreactivity is observed in developing podocytes (P) and mesangial cells (M) (panel B). VE-PTP expression is also observed in the cells dispersively distributed around developing glomeruli (white arrowheads in panel A, black arrowheads in panel B). G, developing glomerulus; VC, vascular cleft. Scale bar, 50 μm in A; 20 μm in B.

**Fig 11:**
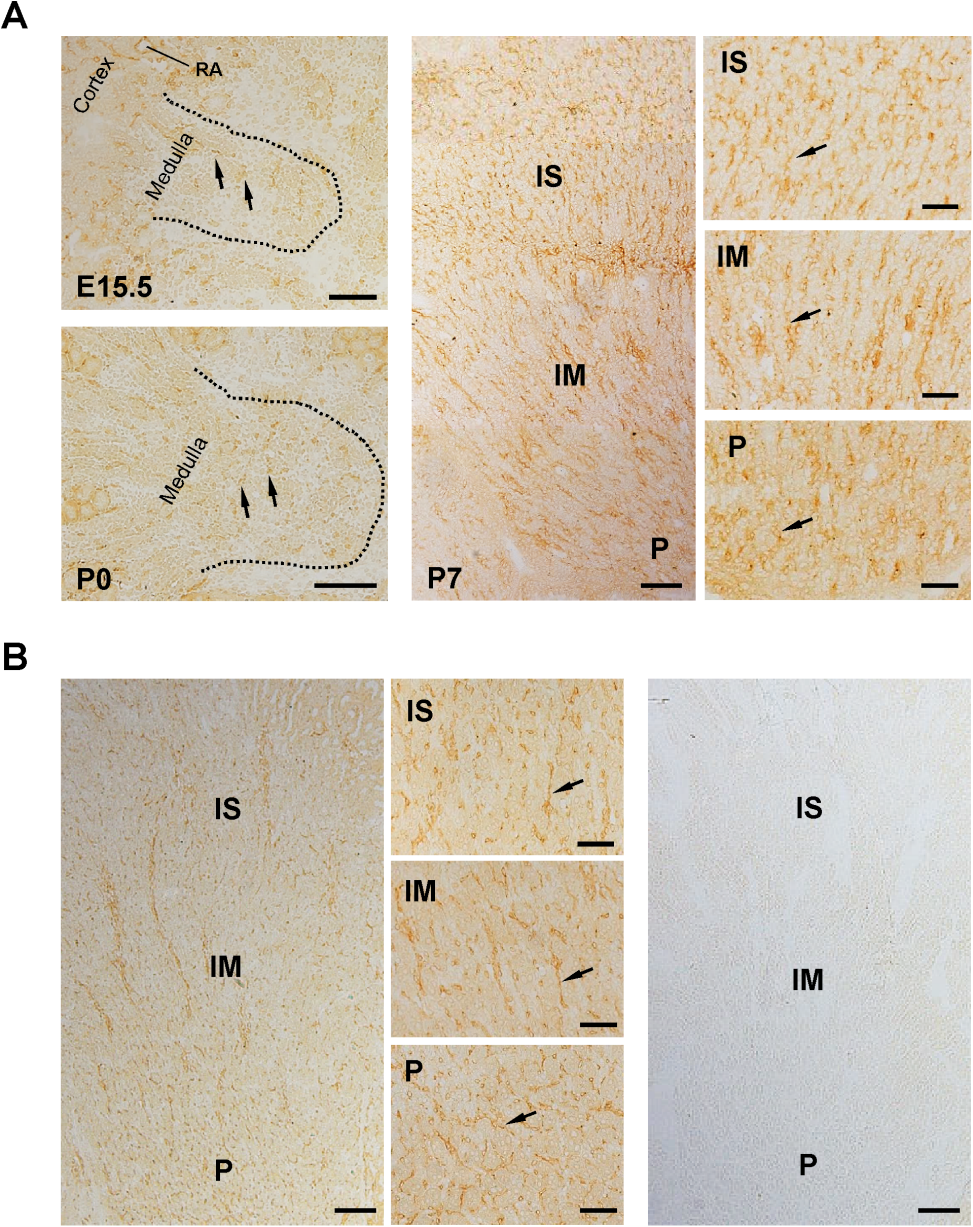
Immunolocalization of VE-PTP in developing and adult renal medulla. **(A)** VE-PTP expression in E15.5, P0, and P7 kidneys was examined by immunohistochemistry using anti-VEPTP rat monoclonal antibody and ABC method as described in the “Materials & Methods”. VE-PTP-expressing cells (arrows) are dispersively distributed in medulla of E15.5 and P0 kidneys (left panels). Its connectivity suggests the formation of medullary vessels by these cells. In P7 kidney (middle and right panels), VE-PTP immunoreactivity is observed in medullary vessels (arrows) distributed along the tubules. Definite VE-PTP expression is not observed in tubular components. IS, inner stripe of outer medulla; IM, inner medulla; P, papilla; RA, renal artery. Scale bar, 100 μm in left and middle panels; 50 μm in right panels. **(B)** VE-PTP expression in adult mouse kidney was examined by immunohistochemistry as in (A). VE-PTP immunoreactivity is observed in vessels (arrows) distributed along the medullary tubules, while obvious VE-PTP expression is not observed in tubular components. Right panel shows the immunostaining without the 1^st^ antibody. IS, inner stripe of outer medulla; IM, inner medulla; P, papilla. Scale bar, 100 μm in left and right panels; 50 μm in middle panels.

**Fig 12:**
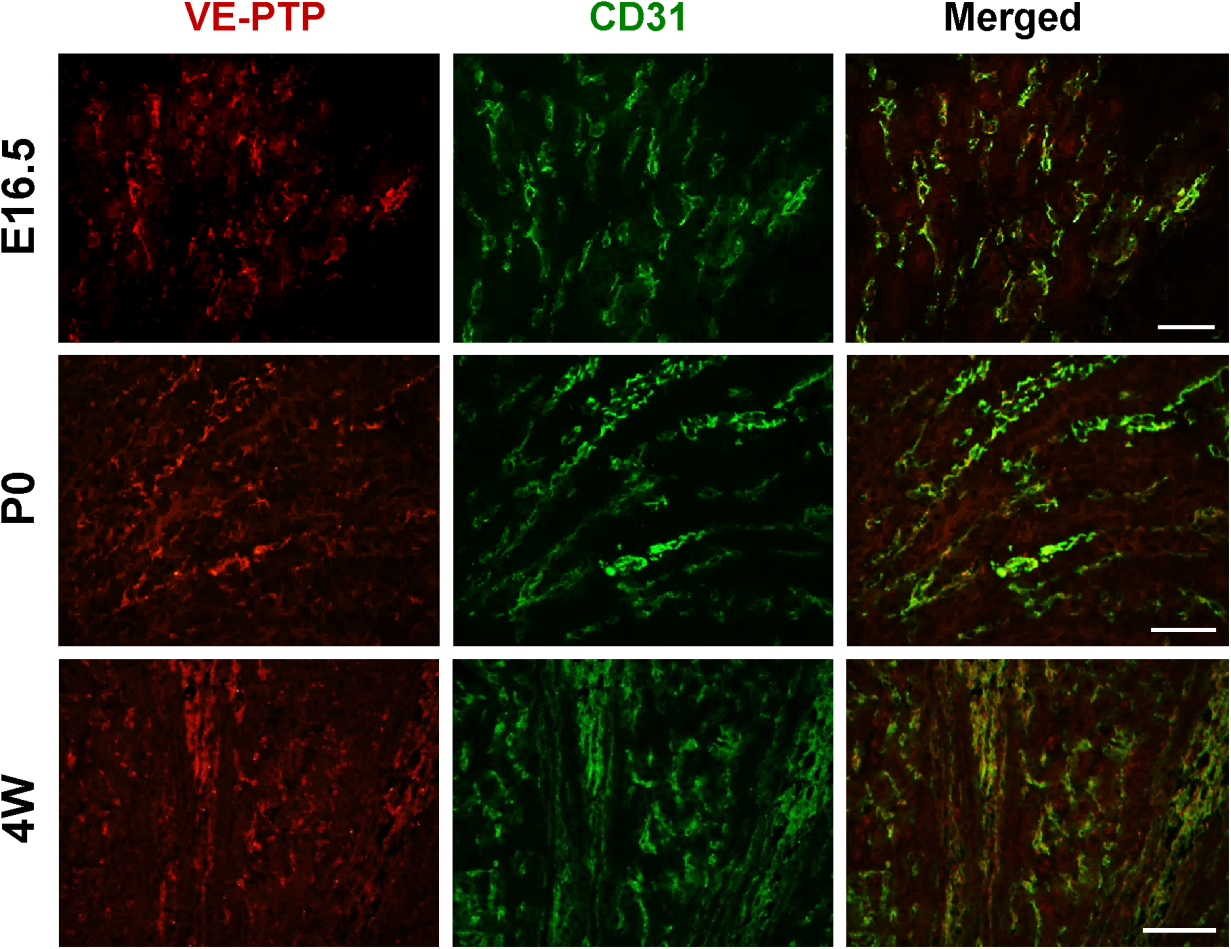
Co-immunostaining of VE-PTP and CD31 in developing and adult renal medulla. Kidney sections from E16.5, P0 and adult (4 weeks) mice were double immunolabeled for VE-PTP (red) and CD31 (green) as described in the “Materials & Methods”. VE-PTP is co-localized with CD31 in renal medulla of developing and adult mice kidneys, indicating its expression in medullary vessels. Scale bar, 50 μm.

In order to evaluate the expression of VE-PTP and PTPμ proteins in developing and adult mouse kidneys, we next conducted immunofluorescence and immunohistochemistry studies using specific antibodies. Endothelial localization of these RPTPs was assessed by co-immunostaining with CD31 or VEGFR2. In embryonic and postnatal kidneys, VE-PTP immunoreactivity was observed in the endothelium of developing renal arterial vessels and glomeruli, and its expression increased as the vasculature matured (**Figs 8A through 8C, 8E, 8F and 9**). VE-PTP expression was also observed in the cells that are distributed around developing glomeruli and tubules in embryonic kidneys (arrowheads in **Fig 8E**, arrows in **Fig 9**, arrowheads in **Fig 10**, arrowheads in **S5 Fig**). These cells expressed VEGFR2 as well as CD31 (**S5 Fig**). Their dispersive distribution and endothelial receptor expression indicate that these are endothelial progenitors that participate in renal vasculogenesis [28, 29]. VE-PTP was also expressed in peritubular capillaries in postnatal kidneys (**Figs 8F and 9**). In the medulla of developing mouse kidneys, VE-PTP expression was observed in medullary vessels (**Fig 11A**) and its distribution overlapped well with CD31 immunolocalization (**Fig 12**), indicating the absence of VE-PTP immunoreactivity in medullary tubular cells. VE-PTP expression was limited in veins in developing kidneys (**Fig 8A through 8C, 8E and 8F**).

The **Fig 10**shows VE-PTP expression during the development of glomerulus. In developing glomeruli, VE-PTP was expressed in vascular clefts (arrows) of S-shaped glomeruli, and its expression increased as glomerular development proceeded (**Fig 10**). VE-PTP expression overlapped well with CD31 immunolocalization (**Fig 10A**) and its immunoreactivity was not observed in mesangial cells and podocytes during glomerular development (**Fig 10B**). The expression of VE-PTP in early glomerular endothelial cells that are present in vascular clefts of S-shaped glomeruli was limited as compared with VEGFR2 expression of these cells (**S5 Fig**).

In adult kidneys, VE-PTP immunoreactivity was observed in the endothelium of renal arterial vessels, afferent arterioles, glomeruli (**Figs 8D, 8G and 9, S5 Fig**), and medullary vessels (**Figs 11B and 12**), whereas its expression was limited in peritubular capillaries and veins (**Figs 8D, 8G and 9**). Although VE-PTP promoter activity was observed in medullary tubular segments (**Fig 1B**), significant immunoreactivity of VE-PTP was not observed in medullary tubules (**Figs 11B and 12**).

**Fig 13:**
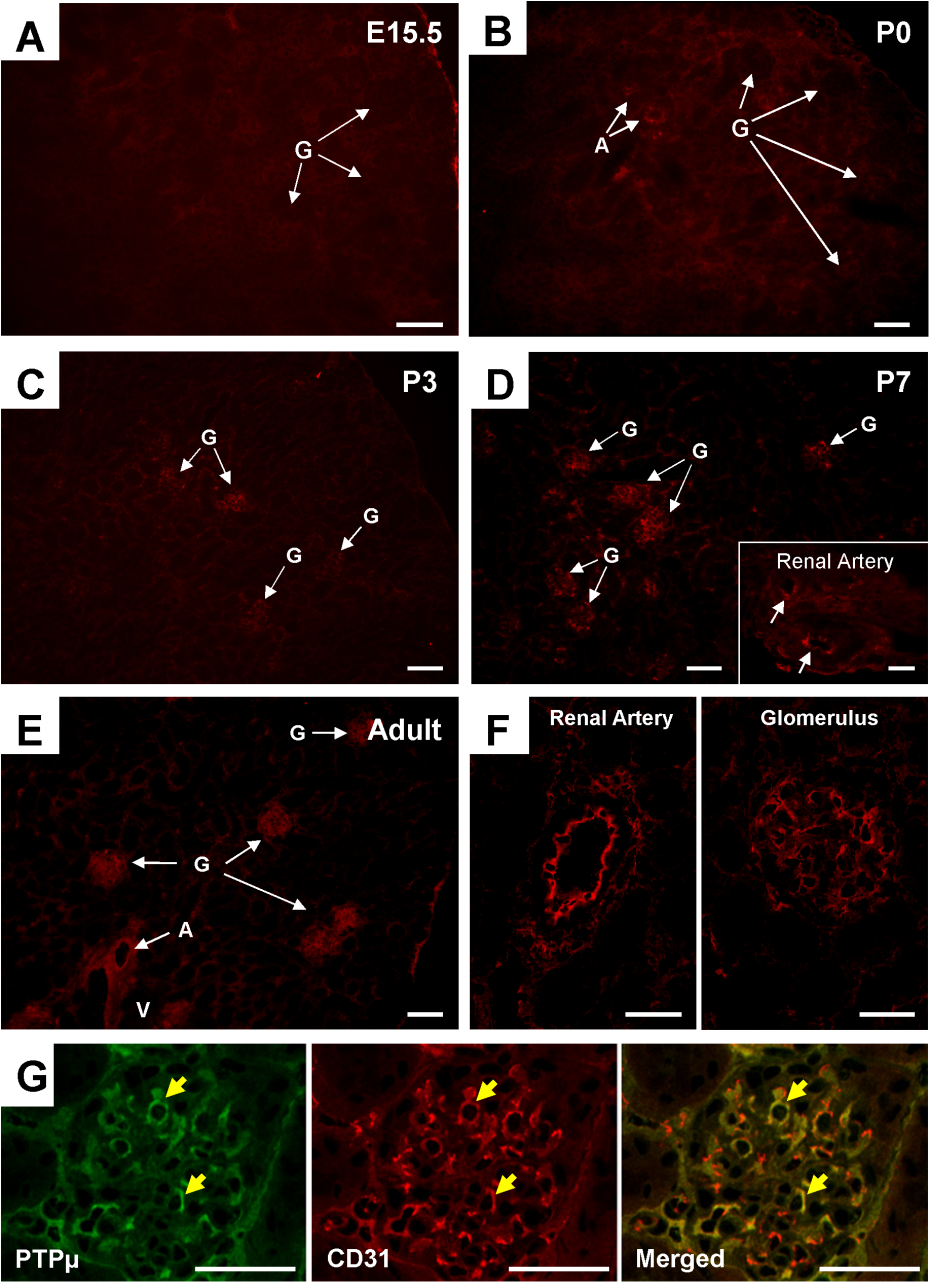
PTPμ expression in developing and adult mouse vasculature. **(A though F)** PTPμ was visualized in developing and adult mice kidneys by immunofluorescence staining using anti-PTPμ mouse monoclonal antibody. PTPμ immunoreactivity is relatively limited in developing kidneys at E15.5 (panel A) and P0 (panel B) stages. Weak immunoreactivity is observed in large renal arterial vessels (A) in P0 kidney. At P3 (panel C) and P7 (panel D), PTPμ is expressed in maturing glomeruli (G) and large renal arterial vessels including renal artery and its branch (an insert in panel D). In adult kidney (panels E and F), PTPμ is expressed in the endothelium of large renal arteries (renal artery to arcuate artery) (A) and glomeruli (G). **(G)** Endothelial localization (yellow arrows) of PTPμ in glomerulus was confirmed by co-immunostaining of PTPμ and CD31. A, arterial vessel; G, glomerulus; V, vein. Scale bar, 60 μm in A, B, C, D, E, and G; 30 μm in F.

The immunolocalization of PTPμ was also examined by immunofluorescence. Although PTPμ promoter activity was observed in embryonic renal vasculature (**Fig 5A through 5C**), evident immunoreactivity was not observed in these kidneys (**Fig 13A**). In neonatal kidneys, immunoreactivity of PTPμ was observed in the endothelium of large renal arterial vessels (**Fig 13B**). In P3 kidneys, PTPμ immunoreactivity was also observed in glomeruli (**Fig 13C**) and the immunoreactivity in these renal vasculatures was increased in P7 kidneys (**Fig 13D**). In adult kidneys, PTPμ immunoreactivity was observed in the endothelium of large renal arterial vessels (renal artery to arcuate artery) and glomerulus (**Fig 13E and 13F**). Endothelial localization of PTPμ in glomerulus was confirmed by co-immunostaining of PTPμ and CD31 (**Fig 13G**). Obvious PTPμ immunolocalization was not noted in interlobular arteries and arterioles and other renal components including medullary vessels and tubules in developing and adult mouse kidneys. Thus, a discrepancy was observed between PTPμ promoter activity and immunolocalization. Recently, it was shown that several forms of PTPμ are processed through proteolysis [16]. This finding suggests that some PTPμ forms cannot be immunohistochemically detected with this antibody. Therefore, we addressed this issue by Western blot analysis. As shown in **S6A and S6B Fig**, the expression of a full-length form of PTPμ was limited in adult mouse kidneys, especially in the renal medulla. Furthermore, we noted that a full-length form of PTPμ is increased on kidney development (**S6C Fig**). These findings suggest that PTPμ immunoreactivity observed in this study may reflect only the full-length PTPμ expression and other PTPμ forms may not be detected with this antibody. Hence, we also tested other PTPμ antibodies (eight antibodies) that were available for us; however, none of them worked sufficiently well for kidney tissue immunostaining.

## Discussion

The present study investigated for the first time the expression of two endothelial RPTPs, VE-PTP and PTPμ, in developing and mature renal vasculature. Both VE-PTP and PTPμ were abundantly expressed in arterial and glomerular vasculatures in adult mouse kidneys, while their expression was limited in peritubular capillaries and veins. A similar expression pattern was observed in developing kidneys, yet VE-PTP was also expressed in developing peritubular capillaries. The endothelial cells in arterial and glomerular vasculatures have to cope with high blood pressure (tensile load) and blood flow (shear stress); therefore, tighter endothelial cell-cell adhesion would be required to maintain the integrity of endothelial layer and barrier function. VE-PTP and PTPμ were shown to interact with VE-cadherin cell-adhesion complex and strengthen endothelial cell-cell adhesion by dephosphorylating VE-cadherin, γ-catenin, or p120 catenin [9, 14, 15, 22, 23]. Furthermore, the ectodomain of PTPμ interacts homophilically and mediates endothelial cell-cell adhesion [18–20]. Thus, there is substantial evidence that these RPTPs strengthen endothelial cell-cell adhesion. This would explain their arterial and glomerular expression. On the other hand, shear-induced stresses imposed on the apical surface of endothelial cells are transmitted to endothelial cell-cell contacts via the cytoskeleton, leading to reorganization of adherens junction and the alignment and reorientation of endothelial cells [30]. Koop et al. have shown that flow-induced dilatation in mesenteric resistance arteries is impaired in PTPμ -/- mice [31]. Mantilidewi et al. demonstrated that VE-PTP accumulates at the downstream edge of the cells relative to the direction of flow and VE-PTP knockdown blocks endothelial cell elongation induced by shear stress [32]. Collectively, these findings suggest that VE-PTP and PTPμ play an important role in maintaining endothelial integrity and mediating shear stress-induced endothelial changes in renal arterial and glomerular vasculatures.

Of interest, both VE-PTP and PTPμ were expressed in medullary capillaries, while their expression was limited in peritubular capillaries in adult kidneys. Although evident PTPμ immunoreactivity was not observed in medullary capillaries, perhaps due to low-level expression of the full-length form of PTPμ, given the fact that medullary capillaries are aligned in more linear shape, this finding suggests that tighter endothelial cell-cell contacts are required for the formation of linear-shape capillaries. In the process of vascular development, endothelial cell-cell contacts need to become tighter for maturation and stabilization of the vasculature. This would be why VE-PTP or PTPμ expression becomes evident on maturation of arterial and glomerular vasculatures, yet mechanisms of this remain to be elucidated. In this context, it is of note that PTPμ surface expression was shown to be up-regulated with increasing cell density, while it is rapidly cleared from the cell surface in the absence of cell-cell contacts [33]. This may explain the low-level expression of full-length PTPμ in developing renal vasculature.

VE-PTP was shown to be associated with angiopoietin receptor Tie-2 and negatively regulate its activity and control endothelial vascularization, vascular maturation and stabilization, and vessel size [10, 11]. In support of this function, the developmental expression pattern of VE-PTP was correlated with the reported Tie-2 expression in developing mouse kidneys; Tie-2 is expressed in arterial endothelial cells, interstitial cells surrounding nephron precursors that generate glomeruli and tubules, and developing peritubular capillaries [34, 35]; Tie-2 is limited in vascular clefts of S-shaped glomeruli as compared with VEGFR2 and its expression increases as glomerular development proceeds [34, 35]; In the medulla, Tie-2 is expressed in spindle-shaped cells that form a loose vessel network at embryonic stage and in capillaries aligned alongside tubules and vasa recta at postnatal stage [34]. Given the fact that Tie-2 ligands, angipoietin-1 or angiopoietin-2, are expressed in the tissue compartments that are associated with these vessels [34], this finding suggests that VE-PTP regulation of angiopoietin-Tie2 signaling play an important role in the formation of these renal vasculatures. In this context, it is of note that a recent study demonstrated that VE-PTP dephosphorylates VEGFR2 in an angiopoietin-1/Tie-2-dependent manner, reducing VE-cadherin phosphorylation, and controls endothelial cell polarity and lumen formation [36]. VE-PTP may play an important role in the morphogenesis of renal vasculature. Although VE-PTP is expressed in VEGFR2-expressing endothelial precursors that are dispersively distributed in metanephric mesenchyme [28, 29], given the fact that vasculogenesis occurs normally in VE-PTP deficient mice [6, 7], it would be less likely that VE-PTP mediates renal vasculogenesis.

VE-PTP is down-regulated on maturation of peritubular capillaries, while Tie-2 is expressed in these vessels [37]. This finding suggests that VE-PTP regulation of Tie-2 signaling is required for the formation of peritubular capillaries but not for the maintenance of these vessels. In this context, it is noteworthy that mice deficient in angiopoietin-2, a context-dependent antagonist for Tie-2, exhibit dysmorphogenesis of cortical peritubular capillaries accompanied by an increase in Tie-2 phosphorylation [38]. The regulation of Tie-2 activity by VE-PTP may be critical for the morphogenesis of peritubular capillaries.

In addition to vascular expression, the present study suggested a role of these RPTPs in medullary tubules. Although these tubular expressions were not detected in our immunostaining, further investigation would be required on this subject, including the forms of VE-PTP or PTPμ that are expressed in medullary tubular cells and the functions of these RPTPs in tubular epithelial cells. In this context, it is of note that VE-PTP promoter activity (β-galactosidase activity) is not observed in renal tubules in VE-PTP heterozygous mice [6] in which a different exon is targeted (Vestweber D, unpublished observation).

In conclusion, our study suggested important roles of VE-PTP and PTPμ in the development and maintenance of renal vasculature. Further investigations would be required to determine the roles of these RPTPs in renal vasculature, including a conditional knockout study and pharmacological manipulation of VE-PTP. Also, it will be important to identify the functional ligand for VE-PTP and investigate its expression in developing and adult kidneys to understand the VE-PTP regulation of renal vasculature. PTPμ -/- mice were viable and showed no aberrant phenotype under normal circumstances [17]. Nevertheless, its role in kidney disease remains unknown. We also noted that efferent arterioles exhibit strong PTPμ promoter activity; however, its role is currently unknown. These issues should be addressed in future studies.

## Supporting information

S1 Fig VE-PTP and PTPμ promoter activity in thick-ascending limbs of Henle and collecting ducts. **(A)** Immunohistochemistry for Tamm-Horsfall protein (THP, brown) or histochemistry of the lectin from *Dolichos biflorus agglutin* (DBA, brown) were superimposed on β-galactosidase histochemistry of adult VE-PTP^tlacZ/+^ mice kidneys using rabbit anti-human Tamm–Horsfall glycoprotein antibody (Biomedical Technologies, Stoughton, MA) and the biotinylated secondary antibody or biotin-conjugated DBA lectin (1:400; Vector Laboratories Inc., Burlingame, CA) as described previously (J Am Soc Nephrol 12: 2673–2682, 2001). The bindings of anti-THP antibody and DBA lectin were visualized using VECTASTAIN ABC System (Vector Laboratories). Scale bar, 50 μm. Note: VE-PTP transcription is observed in segments of THP-expressing thick ascending limbs of Henle (red arrows) and subpopulations of collecting ducts labeled with DBA lectin in papilla (yellow arrows), while its expression is absent in the collecting ducts in ISOM. **(B)** Immunohistochemistry for THP or DBA lectin histochemistry were superimposed on β-galactosidase histochemistry of adult PTPμ^tlacZ/+^ mice kidneys as in (A). Scale bar, 50 μm. Note: PTPμ promoter activity is observed in collecting ducts in ISOM and papilla, which are labeled with DBA2 lectin (yellow arrows), while its expression is absent in THP-expressing thick ascending limbs of Henle.
(TIF)

S2 Fig β-galactosidase histochemistry of wild-type mouse kidney. Wild-type kidney at postnatal day 3 stage was subjected to β-galactosidase histochemistry. β-galactosidase histochemistry was carried out with the same protocol for PTPμ^tlacZ/+^ kidneys. Non-specific β-galactosidase activity is observed in tubules of outer stripe of outer medulla. Similar non-specific β-galactosidase activity was also observed in adult wild-type mouse kidney (data not shown). Scale bar, 50 μm.
(TIF)

S3 Fig β-galactosidase histochemistry of VE-PTP^tlacZ/+^ mouse kidney at E13.5. VE-PTP promoter activity is observed in ingrowing renal arterial vessels (A) and juxta-medullary glomeruli (G). VE-PTP promoter activity is also observed in the cells that are distributed around arterial vessels (arrows) and mesenchymal condensates (arrowheads). Scale bar, 50 μm.
(TIF)

S4 Fig VE-PTP promoter activity in renal medulla at P7. VE-PTP^tlacZ/+^ mouse kidney at the age of P7 was subjected to β-galactosidase histochemistry. VE-PTP promoter activity is observed in segments of medullary tubules (arrows) as well as in medullary vessels (V). Scale bar, 100 μm (top); 50 μm (middle and bottom).
(TIF)

S5 Fig Co-immunostaining of VE-PTP and VEGFR2 in E17.5 and adult mice kidneys. Kidney sections from E17.5 and adult mice were double immunolabeled for VE-PTP (red) and VEGFR2 (green) using anti-VE-PTP rat monoclonal antibody (Clone 109.3, 10 μg/ml) and FITC-conjugated anti-Flk1 (VEGFR2) rat monoclonal antibody (5 μg/ml; BD Biosciences). The early glomerular endothelial cells (arrows) that are distributed in vascular clefts of S-shaped glomeruli show the limited VE-PTP expression compared with mature glomerular endothelial cells. VE-PTP immunoreactivity is also observed in the VEGFR2-expressing endothelial cells (arrowheads) that are distributed around the developing glomeruli. A, arterial vessel. Scale bar, 20 μm in A and B.
(TIF)

S6 Fig Expression of PTPμ in adult and developmental mouse kidneys. **(A and B)** Kidney and lung tissues were isolated from adult mice and lysed in RIPA buffer [50 mM Tris/pH 8.0, 150 mM NaCl, 1.0% Triton X-100, 0.5% sodium deoxycholate, 0.1% SDS, a proteinase inhibitor cocktail (Roche Diagnostics, Indianapolis, IN)]. The clarified tissue lysates (100 μg) were separated on 6% SDS-polyacrylamide gel under the reducing conditions, transferred to a membrane, and immunoblotted using an anti-PTPμ mouse monoclonal antibody (clone BK2, Santa Cruz Biotechnology) that recognizes the extracellular segment (MAM domain) of PTPμ. Loading was assessed by re-probing the membrane with anti-β actin antibody (N21, Santa Cruz Biotechnology). The protein of ~200 kDa indicates the full length form (FL) and the ~110 kDa protein indicates the cleaved extracellular domain (E) of PTPμ. NS indicates non-specific signals. **(C)** Kidney tissue lysates were prepared from the mice at the indicated age and subjected to immunoblotting as described above. The expression of full-length PTPμ increases on kidney development.
(TIF)
