# Supplementary figures and images for: Expression of receptor-type protein tyrosine phosphatase in developing and adult renal vasculature
Source: PLoS One. 2017 May 25;12(5):e0177192. doi: 10.1371/journal.pone.0177192 (PMC5444631; doi:10.1371/journal.pone.0177192)

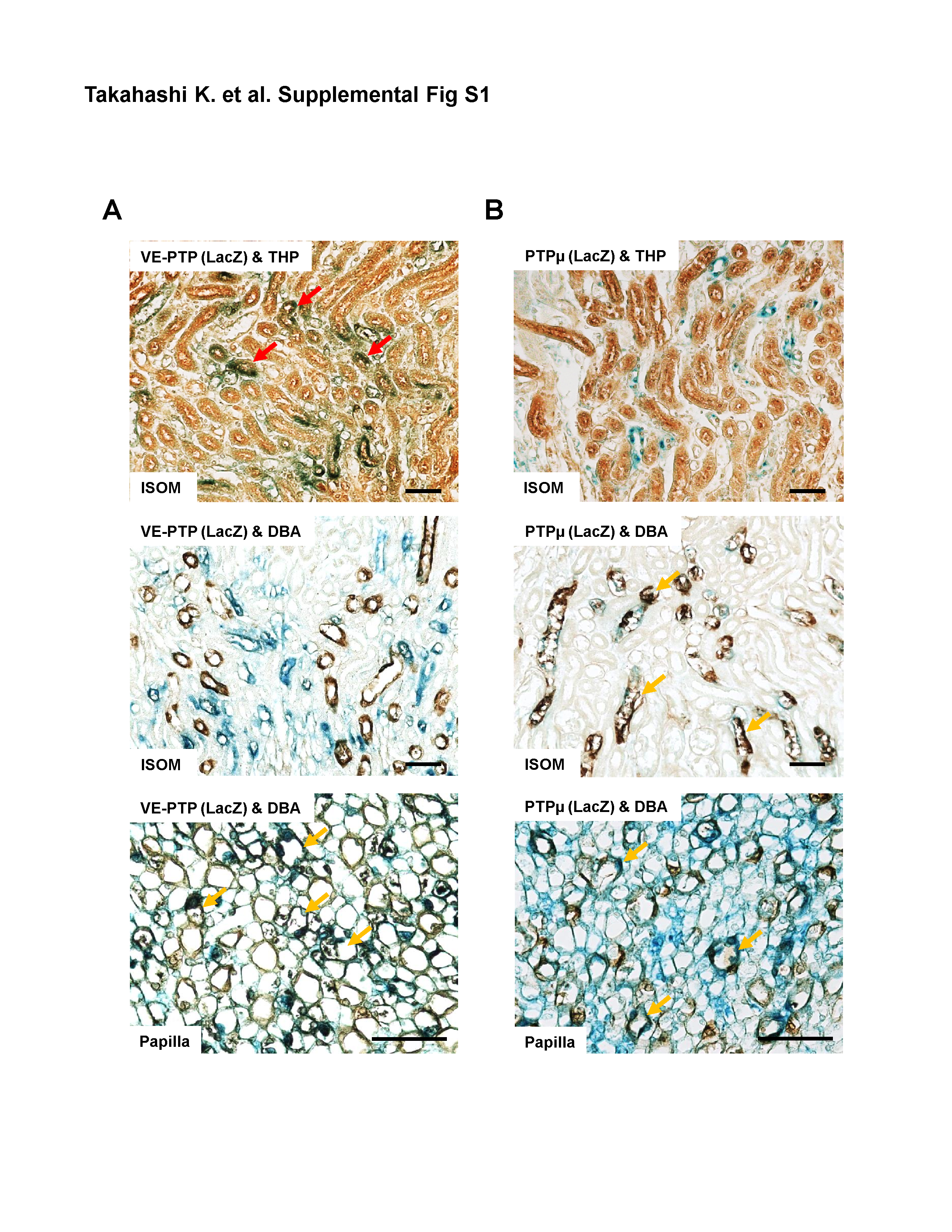

Supplement: S1 Fig — (A) Immunohistochemistry for Tamm-Horsfall protein (THP, brown) or histochemistry of the lectin from Dolichos biflorus agglutin (DBA, brown) were superimposed on β-galactosidase histochemistry of adult VE-PTPtlacZ/+ mice kidneys using rabbit anti-human Tamm–Horsfall glycoprotein antibody (Biomedical Technologies, Stoughton, MA) and the biotinylated secondary antibody or biotin-conjugated DBA lectin (1:400; Vector Laboratories Inc., Burlingame, CA) as described previously (J Am Soc Nephrol 12: 2673–2682, 2001). The bindings of anti-THP antibody and DBA lectin were visualized using VECTASTAIN ABC System (Vector Laboratories). Scale bar, 50 μm. Note: VE-PTP transcription is observed in segments of THP-expressing thick ascending limbs of Henle (red arrows) and subpopulations of collecting ducts labeled with DBA lectin in papilla (yellow arrows), while its expression is absent in the collecting ducts in ISOM. (B) Immunohistochemistry for THP or DBA lectin histochemistry were superimposed on β-galactosidase histochemistry of adult PTPμtlacZ/+ mice kidneys as in (A). Scale bar, 50 μm. Note: PTPμ promoter activity is observed in collecting ducts in ISOM and papilla, which are labeled with DBA2 lectin (yellow arrows), while its expression is absent in THP-expressing thick ascending limbs of Henle. (TIF) [file pone.0177192.s001.tif]

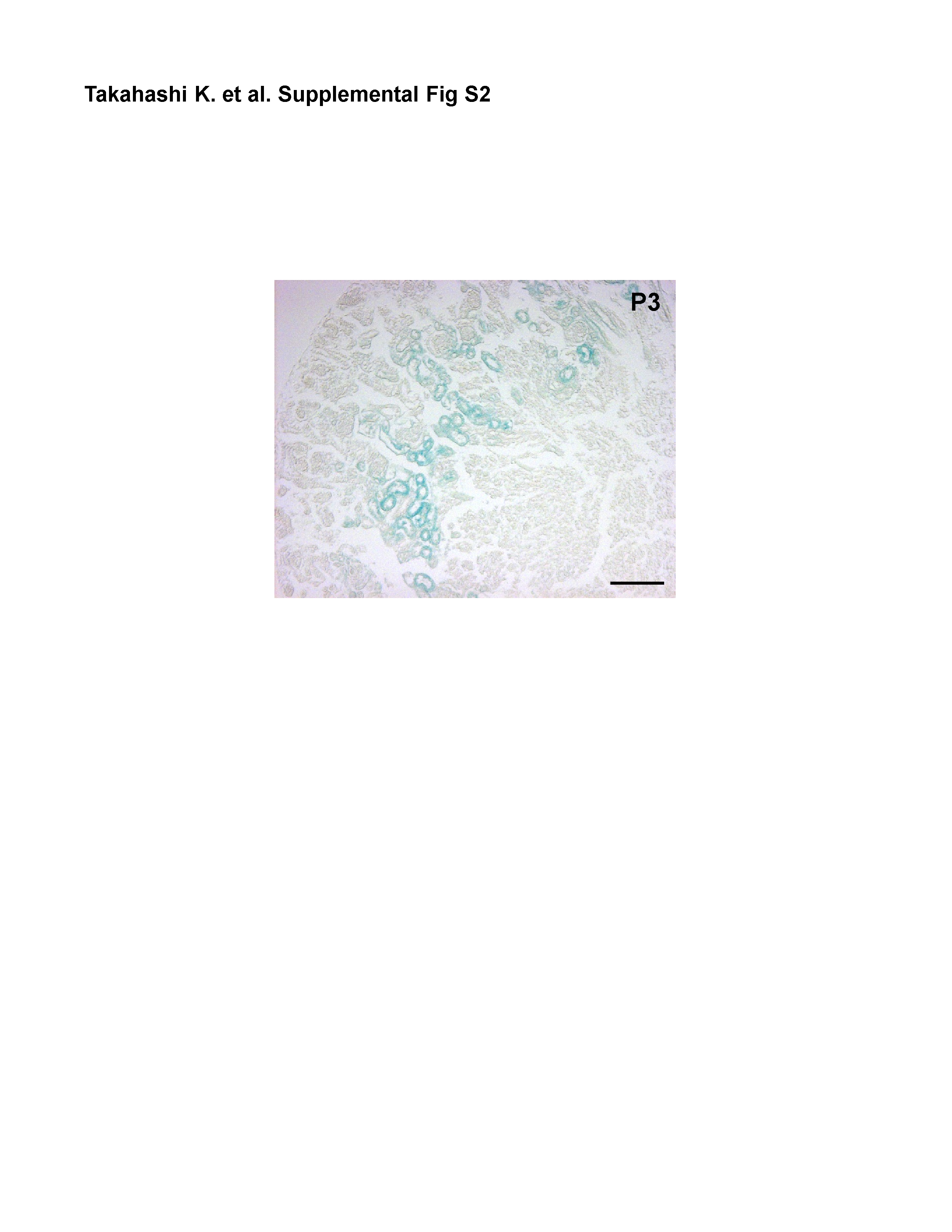

Supplement: S2 Fig — Wild-type kidney at postnatal day 3 stage was subjected to β-galactosidase histochemistry. β-galactosidase histochemistry was carried out with the same protocol for PTPμtlacZ/+ kidneys. Non-specific β-galactosidase activity is observed in tubules of outer stripe of outer medulla. Similar non-specific β-galactosidase activity was also observed in adult wild-type mouse kidney (data not shown). Scale bar, 50 μm. (TIF) [file pone.0177192.s002.tif]

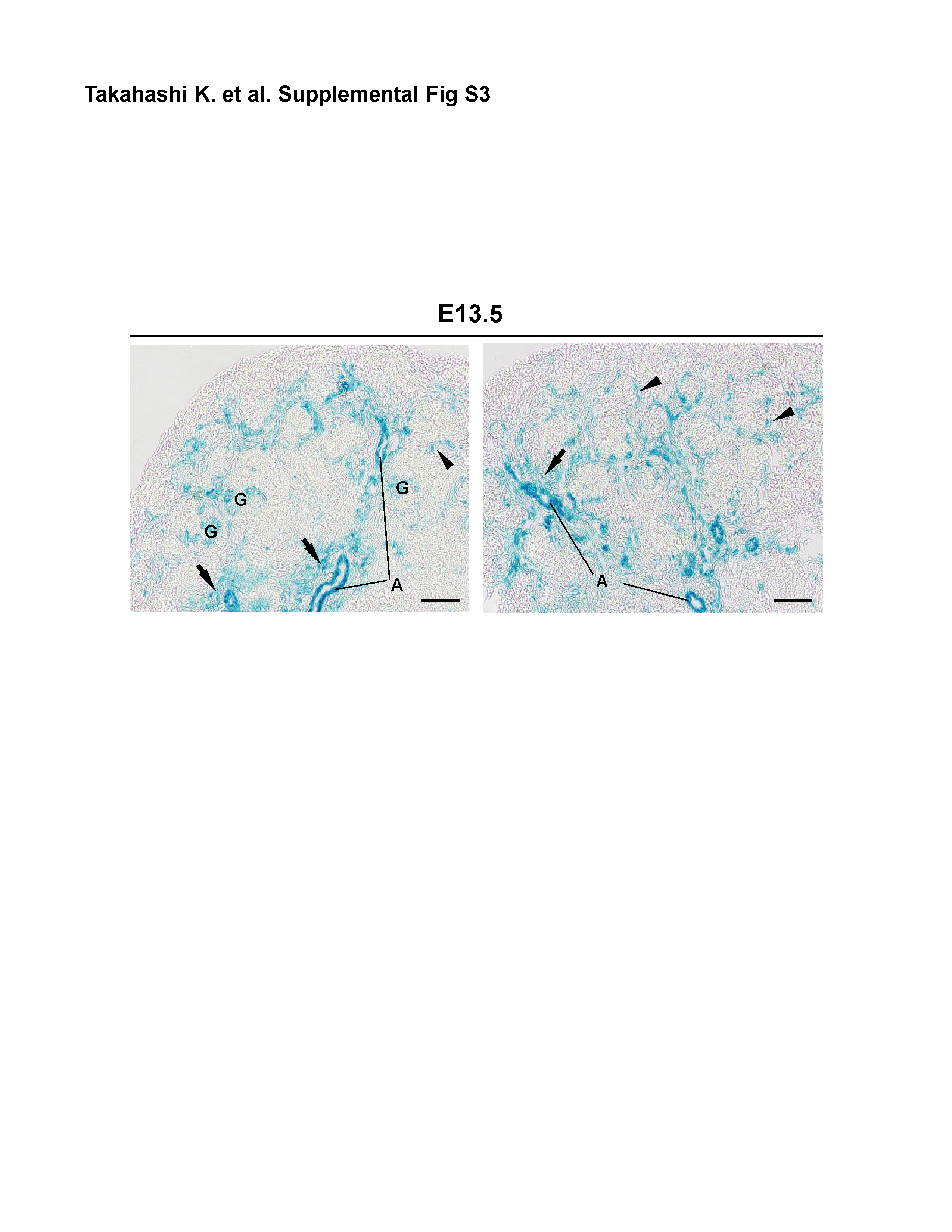

Supplement: S3 Fig — VE-PTP promoter activity is observed in ingrowing renal arterial vessels (A) and juxta-medullary glomeruli (G). VE-PTP promoter activity is also observed in the cells that are distributed around arterial vessels (arrows) and mesenchymal condensates (arrowheads). Scale bar, 50 μm. (TIF) [file pone.0177192.s003.tif]

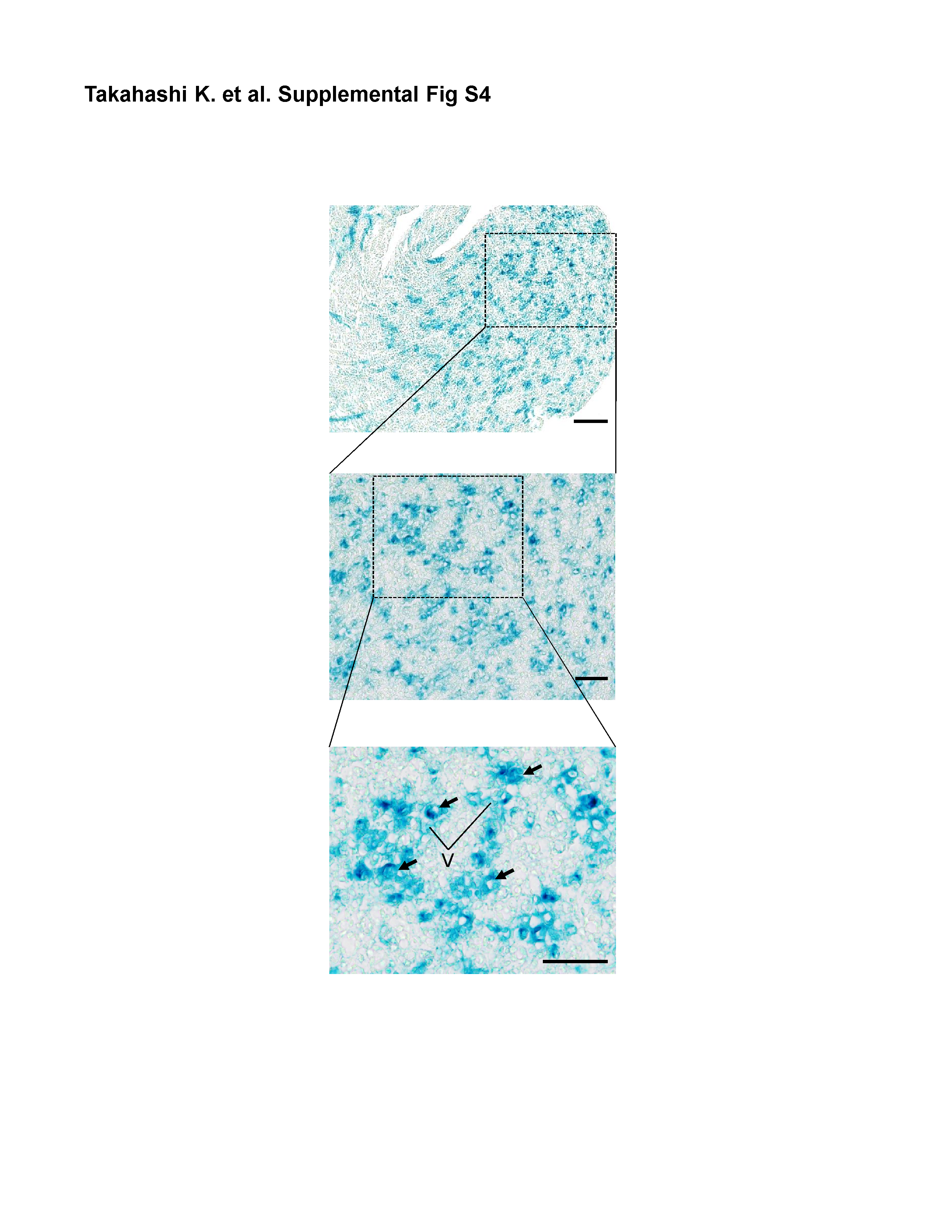

Supplement: S4 Fig — VE-PTPtlacZ/+ mouse kidney at the age of P7 was subjected to β-galactosidase histochemistry. VE-PTP promoter activity is observed in segments of medullary tubules (arrows) as well as in medullary vessels (V). Scale bar, 100 μm (top); 50 μm (middle and bottom). (TIF) [file pone.0177192.s004.tif]

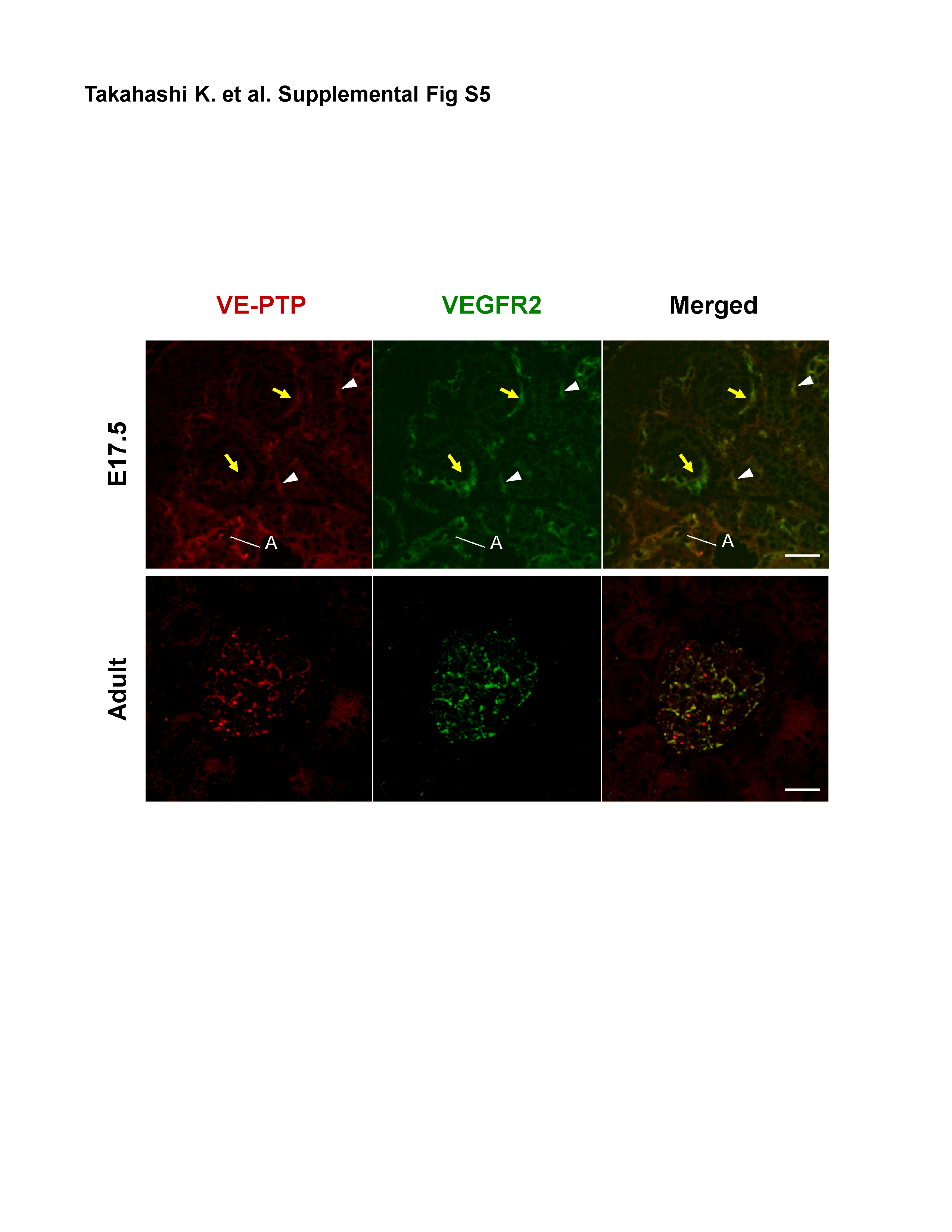

Supplement: S5 Fig — Kidney sections from E17.5 and adult mice were double immunolabeled for VE-PTP (red) and VEGFR2 (green) using anti-VE-PTP rat monoclonal antibody (Clone 109.3, 10 μg/ml) and FITC-conjugated anti-Flk1 (VEGFR2) rat monoclonal antibody (5 μg/ml; BD Biosciences). The early glomerular endothelial cells (arrows) that are distributed in vascular clefts of S-shaped glomeruli show the limited VE-PTP expression compared with mature glomerular endothelial cells. VE-PTP immunoreactivity is also observed in the VEGFR2-expressing endothelial cells (arrowheads) that are distributed around the developing glomeruli. A, arterial vessel. Scale bar, 20 μm in A and B. (TIF) [file pone.0177192.s005.tif]

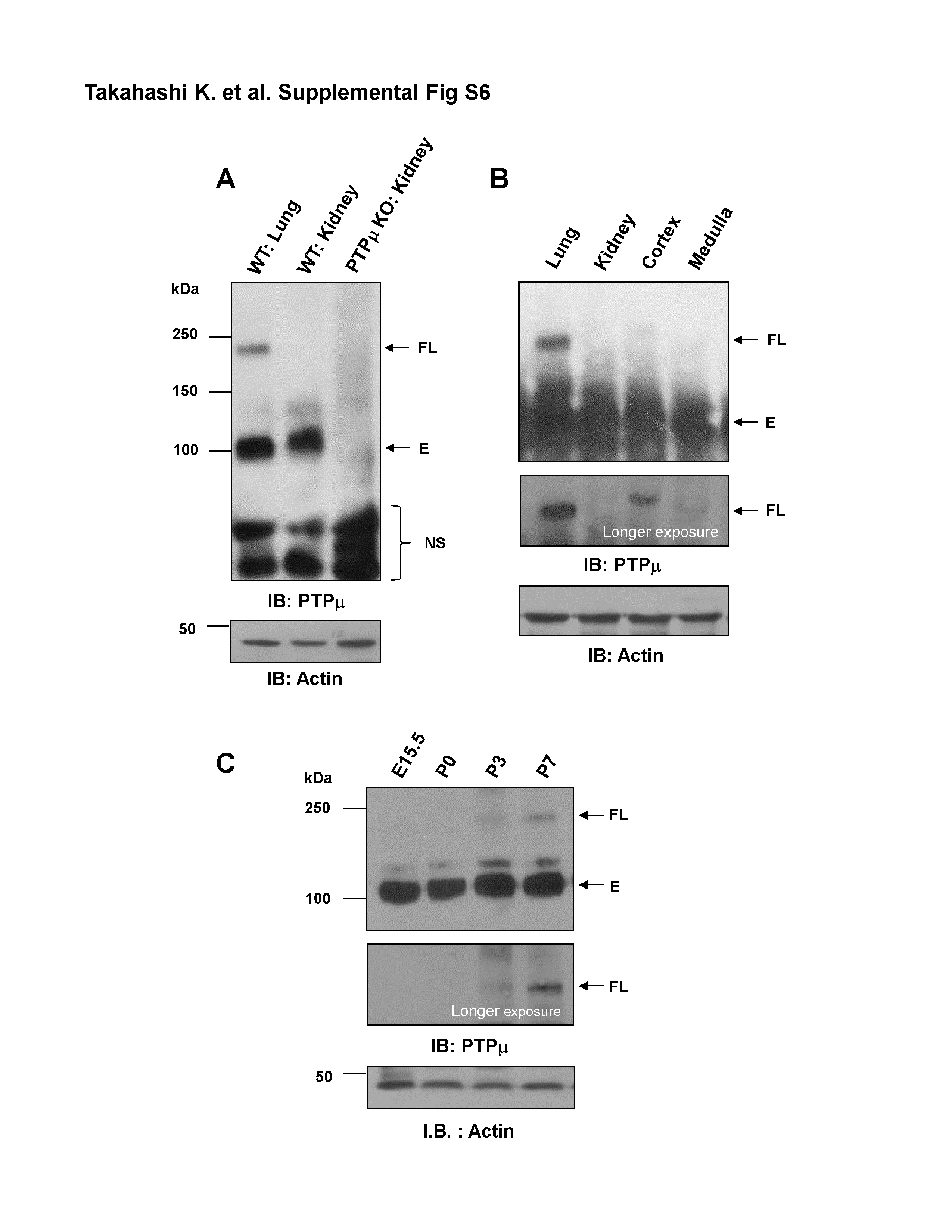

Supplement: S6 Fig — (A and B) Kidney and lung tissues were isolated from adult mice and lysed in RIPA buffer [50 mM Tris/pH 8.0, 150 mM NaCl, 1.0% Triton X-100, 0.5% sodium deoxycholate, 0.1% SDS, a proteinase inhibitor cocktail (Roche Diagnostics, Indianapolis, IN)]. The clarified tissue lysates (100 μg) were separated on 6% SDS-polyacrylamide gel under the reducing conditions, transferred to a membrane, and immunoblotted using an anti-PTPμ mouse monoclonal antibody (clone BK2, Santa Cruz Biotechnology) that recognizes the extracellular segment (MAM domain) of PTPμ. Loading was assessed by re-probing the membrane with anti-β actin antibody (N21, Santa Cruz Biotechnology). The protein of ~200 kDa indicates the full length form (FL) and the ~110 kDa protein indicates the cleaved extracellular domain (E) of PTPμ. NS indicates non-specific signals. (C) Kidney tissue lysates were prepared from the mice at the indicated age and subjected to immunoblotting as described above. The expression of full-length PTPμ increases on kidney development. (TIF) [file pone.0177192.s006.tif]
